# Rational Design of Metal-Doped Graphitic Materials for Enhanced Lithium–Sulfur Batteries

**DOI:** 10.1021/acsnano.5c10546

**Published:** 2025-10-14

**Authors:** Vy Nguyen, Xueyan Lin, Rishav Baranwal, Haiyan Tan, David Wright, Zhaoyang Fan, Bin Wang

**Affiliations:** † School of Sustainable Chemical, Biological and Materials Engineering, 6187 University of Oklahoma, Norman, Oklahoma 73019, United States; ‡ School for Engineering of Matter, Transport & Energy, 7864 Arizona State University, Tempe, Arizona 85281, United States; § Institute of Materials Science, 7712 University of Connecticut, Storrs, Connecticut 06269, United States; ∥ Leroy Eyring Center for Solid State Science, 7864 Arizona State University, Tempe, Arizona 85287, United States; ⊥ School of Electrical, Computer and Energy Engineering, 7864 Arizona State University, Tempe, Arizona 85281, United States; # Max Planck Institute for Sustainable Materials gmbh, Düsseldorf D−40237, Germany

**Keywords:** lithium−sulfur batteries, bond activation, single atom catalysis, DFT, niobium, kinetics

## Abstract

Atomically dispersed metal atoms within graphitic carbon have shown great potential in enhancing the performance of lithium–sulfur batteries (LSBs), though the fundamental principles to guide their rational design remain to be fully established. Here, we report a combined computational and experimental study demonstrating that a group of metals (Ti, V, Mo, and Nb) incorporated into graphitic carbon have promising catalytic properties due to three factors: strong binding with lithium sulfides, reduced redox overpotentials, and low kinetic barriers for Li–S bond activation. In contrast, metals such as Fe and Mn show moderate catalytic behavior, while Ni representing a third group of elements has worse performance. To validate these computational predictions, we synthesized and studied three representative metal elements—Nb, Fe, and Ni—each exhibiting distinct capabilities in binding LiPSs/Li_2_S and catalyzing polysulfide conversion with varying overpotentials and kinetic barriers. Among them, Nb delivered the most exceptional performance, including superior rate capability (679.3 mA h g^–1^ at 5 C), high capacity retention (837.5 mA h g^–1^), and a low capacity decay rate (0.023% per cycle) after 500 cycles at 1 C. This work demonstrates an effective strategy that combines theoretical screening and experimental validation in exploring atomically dispersed metal catalysts for LSBs.

## Graphical abstract

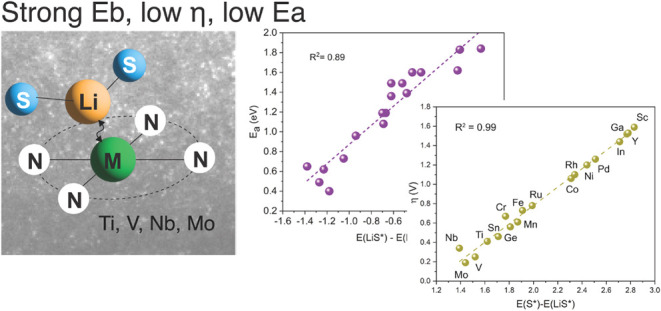

## Introduction

Lithium–sulfur batteries (LSBs), due to their much higher theoretical capacity (1675 mA h g^–1^) and energy density (2600 W h kg^–1^) than conventional lithium–ion batteries (LIBs), have received extensive research interests for applications in electric vehicles, grid energy storage, aviation, and space missions.^1–5^ However, significant challenges impede their deployment, including the low conductivity of the initial S_8_ and final Li_2_S products, the detrimental shuttling effect of lithium polysulfides (LiPSs), a complex 16-electron conversion process, and the sluggish redox conversion kinetics of LiPSs during charge and discharge cycles.^6–10^ Highly porous and conductive carbon materials have been investigated as sulfur hosts to physically confine the sulfur species and improve cathode conductivity.^11^ Nevertheless, the immobilization of polar LiPS species on nonpolar carbon surfaces remains limited, and the stepwise “solid–liquid–solid” multielectron phase transformation process is still constrained due to sluggish redox kinetics.^1,12^

The introduction of heteroatom dopants into carbon materials and various redox mediators/catalysts has been investigated to capture and convert LiPSs.^13–18^ Conventional catalyst materials face challenges like limited charge transfer (i.e., poor conductivity) and/or weak LiPS adsorption capability (i.e., low binding energy).^19^ Moreover, they are typically present as aggregates within the cathode, featuring heavy mass and sizes ranging from tens to hundreds of nanometers, resulting in a low density of active sites. A large amount of these materials is thus required to achieve sufficient catalytic efficiency, which reduces the fraction of sulfur (the active cathode material) and compromises the overall energy density of LSBs. Alternatively, catalysts with atomically dispersed heteroatoms, or so-called single-atom catalysts (SACs), are highly desirable in sulfur cathode design for enhancing access to electrochemically active sites.

Transition metal-based SACs are typically synthesized by coordinating metal atoms on nitrogen-doped graphitic surfaces, forming a unique electronic structure, with a theoretical 100% atom utilization efficiency and abundant active sites compared to conventional bulk metal and nanoparticle catalysts in LSBs.^20–22^ SACs are believed to chemisorb LiPSs and catalyze their redox conversion, thereby improving reaction kinetics by reducing energy barriers, even with low mass loading of SACs within sulfur cathodes.^23,24^ However, the catalytic behavior and ultimate electrochemical performance are highly affected by the transition-metal centers and electronic structures of SACs.^25,26^ Many metal and nonmetal catalytic active sites, such as N, S, V, Fe, Co, Nb, and even dual metal centers have been proposed and shown to improve performance, though it is not straightforward to compare all the work to reveal which metal(s) perform better.^10,23,27–29^ Moreover, despite these efforts, a rational design principle combining both thermodynamics and kinetics for such sites remains to be established.

Here, we report density functional theory (DFT) calculations, through which we propose a few promising metal active centers based on three factors—strong binding energy of lithium sulfides, reduced overpotential of the redox chemistry, and low kinetic barrier for the Li–S bond activation. We conduct comprehensive DFT computations to explore atomically dispersed transition metals (groups 3 to 10 among 3d and 4d metals) and groups 13 and 14 elements (Ga, In, Ge, Sn) anchored onto nitrogen-doped graphene. We evaluate their encapsulation abilities and catalytic activities, and we also delve into the interaction mechanisms between the catalysts and sulfur species from an electronic structure perspective to uncover the underlying patterns in catalytic activity. Among the metals, we find that Ti, V, Nb, and Mo show enhanced LiPS binding (<−3 eV for Li_2_S binding), lower overpotential (less than 0.5 V for the Li–S bond dissociation steps), and lower kinetic barrier (less than 1 eV) for activating the Li–S bonds. This group is followed by the second group of metals (Fe, Mn, Ru, and Cr), which have moderate binding (∼−2 eV for Li_2_S binding), overpotential (0.5–0.8 V), and activation barrier (1.2–1.5 eV). These electrochemical properties also correlate with the intrinsic electronic structure of the metal centers, such as the d–band center, and the stability of key intermediate species, such as LiS. A third group of metals, represented by Ni, was predicted to exhibit the least favorable catalytic activity. Guided by these theoretical insights, we employed a facile dissolution–carbonization method to synthesize a series of atomically dispersed metal (M = Nb, Fe, Ni) atoms anchored on nitrogen-doped porous carbon (M-SA/NC). These catalysts were experimentally evaluated for their catalytic effects on sulfur redox reactions and their overall impact on the LSB performance. Consistent with theoretical expectations, experimental results reveal that Nb-SAC exhibits the most effective capability in capturing polysulfide intermediates and accelerating their redox conversion by lowering the associated energy barrier. LSBs incorporating Nb-SA/NC catalysts delivered impressive electrochemical performance, including a high rate capability of 679.3 mA h g^–1^ at 5 C, excellent capacity retention of 837.5 mA h g^–1^ and a low capacity decay rate of just 0.023% per cycle over 500 cycles at 1 C. Compared to Fe and Ni SACs, the Nb catalyst achieved superior rate capability and long-term stability. This work highlights an effective strategy that integrates theoretical screening with experimental validation to guide the development of high-performance atomically dispersed catalysts for LSBs.

## Results and Discussions

### Adsorption of Li_2_S_*m*_ (*m* = 1, 2, 8) on Metal-SA/NG

The catalysts should exhibit effective adsorption of LiPSs and their facile conversion to insoluble sulfides such as Li_2_S_2_ and Li_2_S (Figure 1a), to prevent their dissolution into the electrolyte. We adopted Li_2_S_*m*_ (*m* = 1, 2, 8) as examples to investigate the adsorption capacity of metal single atoms in nitrogen-doped carbon (M-SA/NC) for LiPS species.^15^ The adsorption configurations of Li_2_S over different metal centers are depicted in Figure S1, showing that the primary interaction at the interface is between the metal centers and sulfur in LiPSs. Note that over Cu and Ag centers, the structures deform upon adsorption of LiS and Li_2_S, respectively (Figure S2), both of which are therefore not included in the following discussions. Figure 1b and c shows the calculated binding energy, *E*_b_, which quantifies the binding strength between LiPSs and the substrates. The dashed lines in Figure 1b and c represent the binding strength between LiPS species and an electrolyte molecule, which is about −0.8 eV. The electrolytes considered in this study were 1,3-dioxolane (DOL) and dimethoxyethane (DME); the interactions between LiPSs and these electrolyte molecules are shown in Figure S3. The binding energy between LiPSs and the substrates should be stronger than that with the electrolyte solvents to mitigate the shuttling effect. Based on the calculation results in Figure 1, most of the metal SACs can provide efficient adsorption strength for LiPSs. Over the single atoms in metals in group 14 (Ge and Sn) and late transition metals in group 10 (Ni and Pd), the values of the two interactions (LiPSs with the solvent and with the metal center) are rather comparable. The metal centers from the early transition metals in groups 3–5 (Sc, Ti, V, Y, Nb, and Mo) exhibit the strongest adsorption. Note that Zr is not included in this calculation due to a convergence issue of the electronic structures, but we expect it should be within this group as well due to its similar oxophilicity.^30^

**Figure 1:**
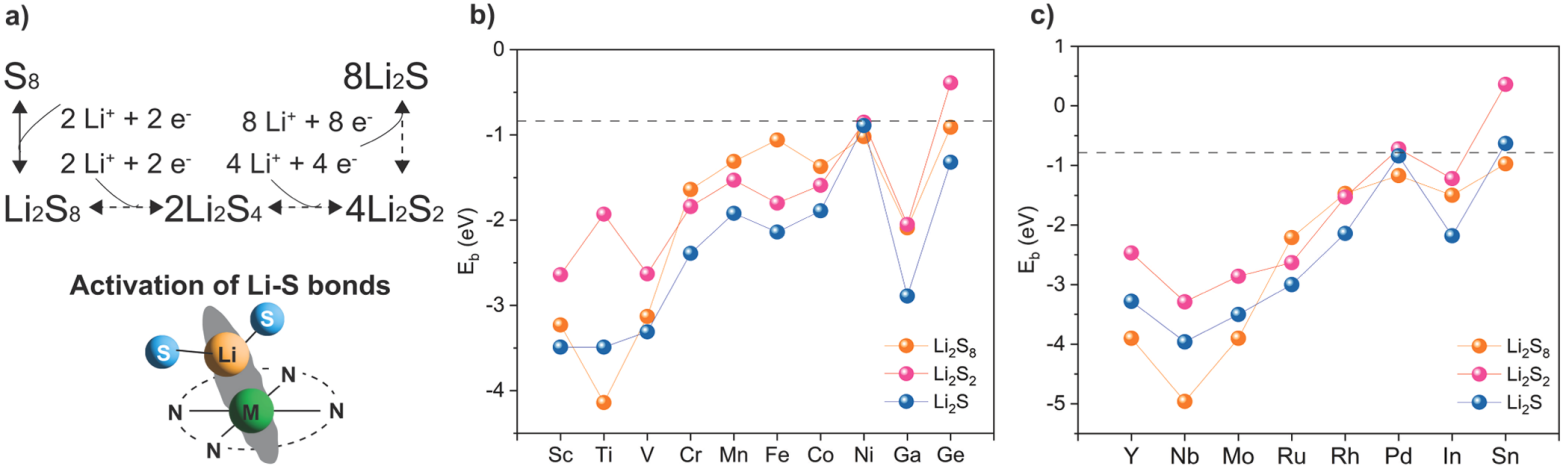
Adsorption of Li_2_S_8_, Li_2_S_2_, and Li_2_S at the metal centers in N-doped carbon (NC). (a) Redox chemistry of LSBs with a few key intermediates illustrated, and it is critical to accelerate the conversion between these species by activating the Li–S bonds, (b) binding energy between Li_2_S_m_ (*m* = 1, 2, 8) and 3d metals, and (c) binding energy between Li_2_S_m_ (*m* = 1, 2, 8) and 4d metals. The dashed lines represent the binding energy between Li_2_S_*m*_ (*m* = 1, 2, 8) and typical electrolyte molecules (DOL and DME). All data points within a series are joined by solid lines to guide the eye.

To provide insights into the observed variation in *E*_b_, we performed an electronic analysis of the metal centers through the projected density of states (PDOS). The metal centers form two covalent bonds and two coordination bonds with the four nitrogen atoms in the first coordination shell.^31^ Using Nb as a representative example for groups 3–5 (Sc, Ti, V, Y, Nb, Mo), Figure 2a demonstrates the PDOS of d orbitals of Nb in Nb-SA/NC. The Nb metal center carries a magnetic moment of 3 μB from DFT calculations due to the three remaining valence electrons as expected. The partially occupied d orbitals of Nb are located close to the Fermi level, enabling robust hybridization with LiPSs. This d orbital hybridization is very different from that of late transition metals; using Pd to represent this group, Figure 2b illustrates that the remaining eight d electrons of Pd^2+^ stay at lower energy levels, and the unoccupied d_*xy*_ orbital is located more than 2 eV above the Fermi level. The large orbital gap between the occupied and unoccupied d orbitals and zero states at the Fermi level results in limited interaction with adsorbates. The implications of this weak interaction are apparent in the configuration of Li_2_S on Pd-SA/NC (Figure S1), where sulfur does not directly interact with the Pd atom on the surface.

**Figure 2:**
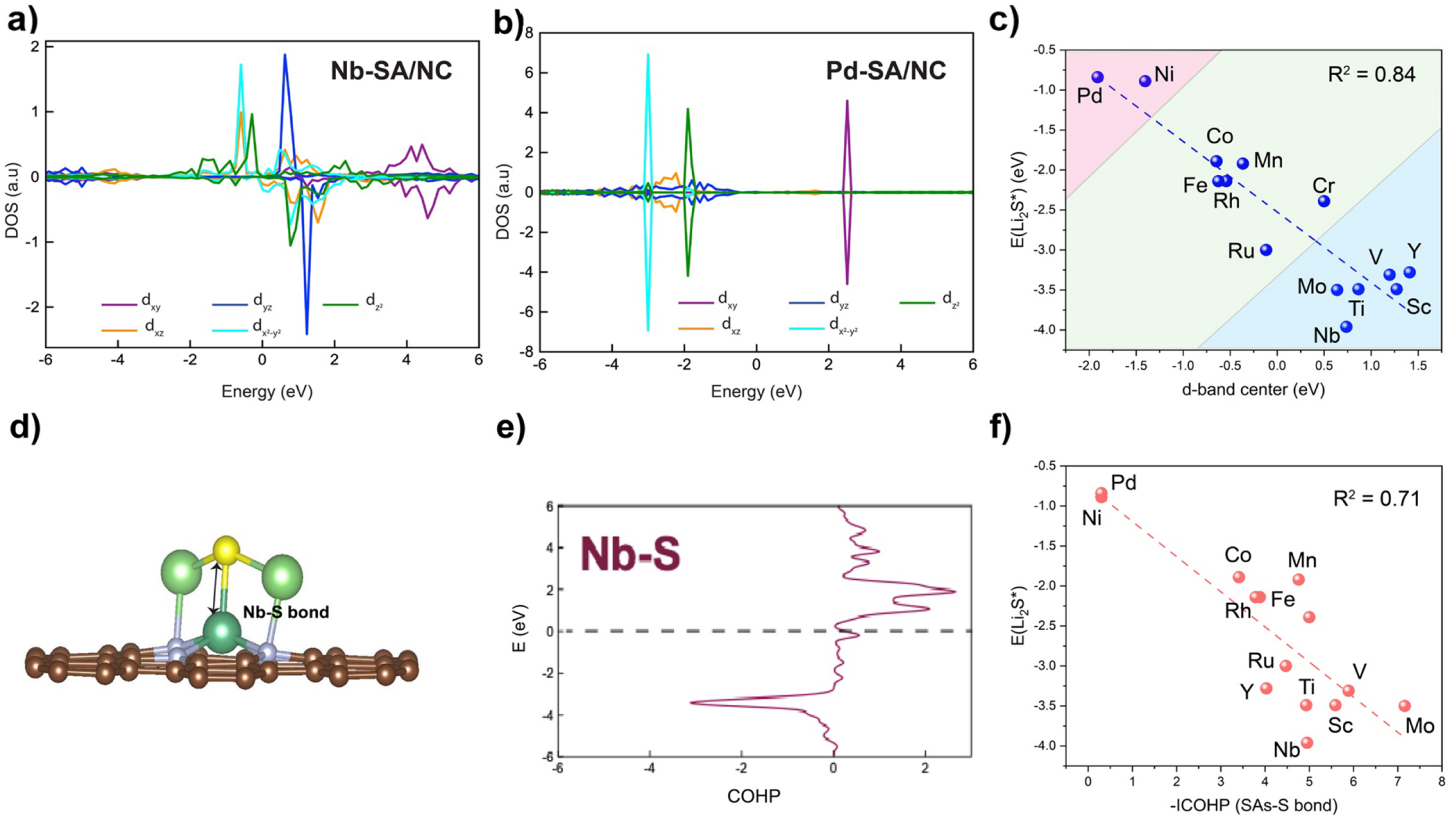
Electronic structural analysis performed using DFT calculations. (a) PDOS of the d orbital of Nb in Nb-SA/NC, (b) PDOS of the d orbital of Pd-SA/NC, and (c) the relationship between the d-band center and the binding energy of Li_2_S on substrates. Positive and negative values in the DOS indicate spin-up and spin-down components, respectively. (d) Atomic structure of Li_2_S on Nb-SA/NC with (e) COHP analysis of the Nb–S bond. (f) The *E*_b_(Li_2_S) versus the strength of the Li–S bond elucidated by the integrated −COHP (−sumICOHP) for Li_2_S adsorption; the dashed line represents the correlation.

The PDOS of all metal-SA/NC surfaces are shown in Figures S4–S6. In groups 3–5, the early transition metals exhibit d orbitals positioned in close proximity to the Fermi level, facilitating strong interactions with adsorbates. In contrast, Figure S6 shows that non-transition metals, such as Ga and In, have partially unoccupied p_*z*_ orbitals close to the Fermi level, resulting in moderate adsorption strength on these metal centers. However, in the case of Ge and Sn, they form a closed-shell electronic structure. That is, the p_*z*_ orbital is fully occupied and situated at approximately −2 eV below the Fermi level, leading to weak adsorption, as shown in Figure 1.

The electronic feature d-band center is widely recognized as a descriptor to assess the strength of the interaction with adsorbates through hybridization. Figure 2c illustrates a correlation between the d-band center of the metal active sites and the binding energy of Li_2_S on surfaces. It is important to note that the binding energy is influenced by other factors, such as the interaction between Li and nitrogen dopants, in addition to the intrinsic properties of the metal active centers; these additional factors likely cause scattering from the linear correlation.

To gain a deeper understanding of the adsorption trend, we further calculated the projected crystal orbital Hamilton population (pCOHP) that considers the overlap and population of adsorbed complexes on SACs. An example of such an analysis of the Nb–S bond is illustrated in Figure 2e, with the atomic structure shown in Figure 2d. The negative COHP signifies the contribution to bonding, while the positive value represents the contribution to antibonding. The integral of the COHP up to the Fermi level (ICOHP) can be used to calculate the overall contribution of the bonding and antibonding states to the bond strength. The primary factor influencing the binding energy of Li_2_S on substrates is the metal–sulfur (metal–S) interaction. As a result, we analyze the bonding and antibonding properties of metal–S bonds, as shown in Figures S7–S9. Moving from left to right across the periodic table for the 3d and 4d metals, the antibonding states shift below the Fermi level, and such shifts lead to increased energy (occupation of the antibonding states) and weakening of the metal–S bond. To quantitatively assess all interactions between the Li_2_S species and the surface, the ICOHP is presented in Table S1 and plotted in Figure 2f. The reasonable correlation between Li_2_S adsorption and the value of the −ICOHP confirms that the metal–S bond dominates the adsorption of Li_2_S. The scattering, as discussed above, should be caused by other interactions at the interface, such as the Li–N interaction. Specifically, the d orbitals of Ni and Pd are located at lower energy levels, leading to weak interaction with adsorbates. On these surfaces, sulfur in Li_2_S does not form covalent bonds with metal centers. Furthermore, the average lengths of Li–S bonds in the adsorbed Li_2_S are significantly longer than those in the isolated Li_2_S molecule (2.08 Å), manifesting strong interaction between LiPSs and the catalysts. The weakening of the Li–S bond implies that metal-SA/NC can effectively activate Li_2_S_*m*_, thereby enhancing its redox chemistry during battery charging and discharging.

### Activity of Metal-SA/NC in Decomposition of Adsorbed Li_2_S

Previous work suggests the last two-electron process (conversion between Li_2_S_2_ and Li_2_S) is the rate-determining step, which may further be divided into two one-electron steps with intermediate LiS.^1,10^ We investigated here the reverse reactions, that is, the oxidation of Li_2_S to S. Similarly, we can write it as a two-electron process shown in eqs 1–2 (or eqs 3–4 if the electrolytes are involved). We determined the potential-limiting step, either Δ*G*_1_ (Li_2_S* to LiS*) or Δ*G*_2_ (LiS* to S*), by calculating the overpotential over the 18 SAC sites based on the thermodynamics of each one-electron process.^10^ We found that among the catalysts studied, the process is limited by the formation of LiS* from Li_2_S on 5 catalysts and by the formation of S* from LiS* on 11 catalysts, while the reaction energy of the two steps is comparable on 2 catalysts (Table S2). Over most catalysts, the differences in reaction energy for these two steps are smaller than 0.58 eV, except for groups 3 and 13 (Sc, Y, Ga, and In) that exhibit a higher reaction energy for the second step by 0.89–1.1 eV than that of the first electrochemical step. This large difference also results in very limited catalytic performance over these four atomic centers.

Figure 3 summarizes the overpotential over these 18 atomic centers; we find that the overpotential can be described linearly as a function of the difference in adsorption energy between S* and LiS*. A smaller difference in the binding energy between S* and LiS* corresponds to a lower overpotential. Specifically, single atoms of early transition metals, such as Mo, V, Nb, and Ti, lie at the lower end of the linear trend, leading to very small absolute overpotentials for the charge transfer processes. However, moderate catalytic performances are observed for single atoms in groups 6–9. The linear trend shown in Figure 3d suggests that the difference in adsorption energy between S* and LiS* can be used as a descriptor to predict the overpotential of the last two one-electron processes. As the metal–LiPS interaction is driven by the metal–S bond, it is anticipated that a similar correlation can be derived from the adsorption difference between S* and Li_2_S*. Indeed, we observe a similar linear trend between this adsorption difference and the overpotential (Figure 3b); however, the data are more scattered. Such a trend is understandable as, for most of the metal centers studied here, the potential-determining step is the reaction shown by eqs 4 and 6 detailed in the methods section. Therefore, correlation of the overpotential with the difference in adsorption energy of S* and LiS* is anticipated. These findings confirm that considering the two-step decomposition of Li_2_S can provide reliable predictions regarding the catalytic performance of metal SACs. Additionally, the presence of the electrolyte solvent does not alter the overall catalytic activity trend of the metal SACs, as the trend remains the same in Figure 3c and e.

**Figure 3:**
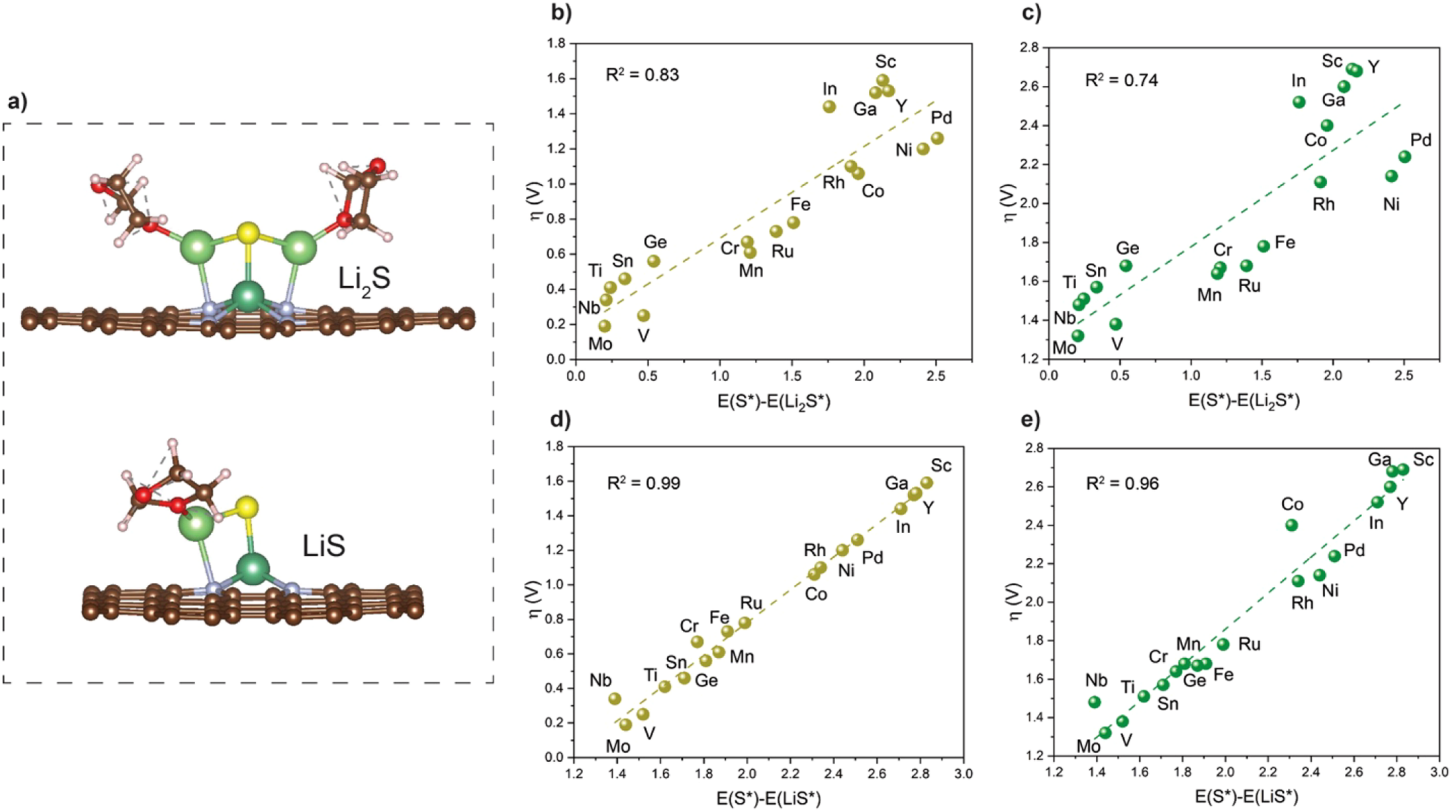
Overpotential of Li_2_S decomposition as a function of the adsorption energy difference of key surface species. (a) Atomic structures of Li_2_S and LiS adsorption at Nb SACs in the presence of solvent molecules. (b,c) Correlation of overpotential with the difference between S* and Li_2_S*. (d,e) Correlation of overpotential with the difference between S* and LiS*. In b and d, the calculations are performed in vacuum, while solvents are included in c and e.

Beyond the overpotential, which is solely based on the thermodynamic driving force of each elementary step, the kinetics of each step should also be calculated explicitly to compare these different metal centers. Figure 4a illustrates the activation barriers for the decomposition of Li_2_S (activation of the Li–S bond) on various substrates. Here, we only focus on the first Li–S bond dissociation in Li_2_S, which should provide fundamental insights into activating the Li–S bond in a variety of LiPSs. We find that the activation energy of the Li–S bond generally increases as the number of valence electrons in 3d and 4d transition metals rises. In Figure 4b, a linear correlation is depicted between the decomposition barrier and the disparity in the binding energy between LiS* and Li_2_S*. That is, a larger difference in binding energy corresponds to a smaller activation barrier. Among the metal centers studied here, the Pd SAC exhibits the highest decomposition barrier (1.83 eV), whereas Nb shows the lowest decomposition barrier (0.40 eV). Transition metals in groups 4–6 (Ti, V, Nb, Mo) and non-transition metals in group 14 (Ge, Sn) have relatively low decomposition barriers (0.40–0.73 eV) and are expected to possess enhanced kinetics for catalytic performance in charge transfer processes that involve Li–S bond activation.

**Figure 4:**
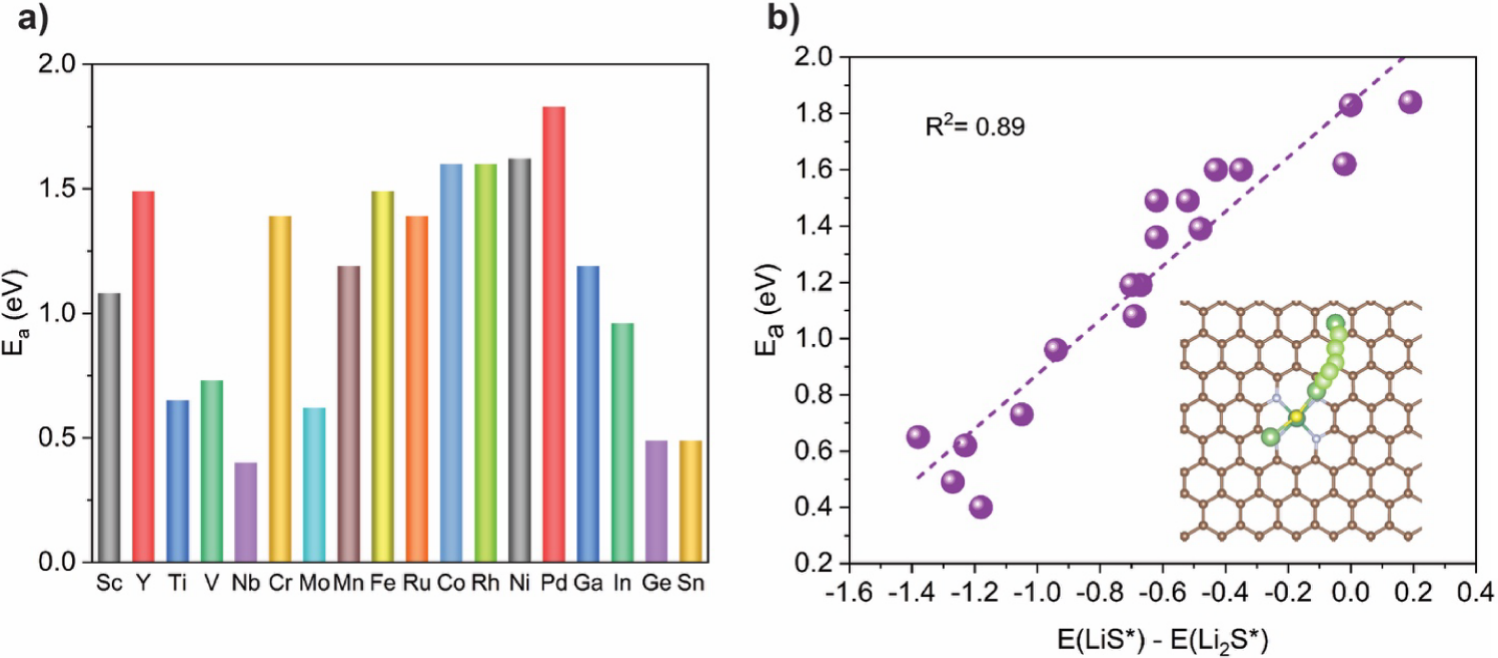
Comparison of the kinetic barrier for activating the Li–S bond. (a) Activation barriers for the decomposition of Li_2_S on various SACs. (b) Activation barriers for the decomposition of Li_2_S as a function of *E*_b_(LiS*) – *E*_b_(Li_2_S*). The dashed line represents the linear relationship. The inset in (b) schematically shows the reaction coordinates during the Li–S bond dissociation of Li_2_S.

By combining all three factors—binding energy of LiPSs at the metal centers, overpotential of the redox chemistry, and kinetic barriers for the Li–S bond activation—now we can theoretically predict the promising SACs using these relatively simplified model structures. Kinetically, Ti, V, Nb, Mo, Ge, and Sn all show activation barriers of 0.5 eV or lower for Li–S dissociation. However, in terms of LiPS binding energy, group 14 (Sn, Ge) does not provide sufficient binding sites for LiPSs. Instead, post-transition metals in group 13 (Ga, In), due to their open-shell electronic structure, exhibit stronger binding energy to reduce the shuttling effect of LiPSs, but they are less favorable in driving the redox chemistry, as shown in the thermodynamic calculations of overpotential (Figure 3) and the kinetic calculations of the Li–S bond activation (Figure 4). That is, metals in groups 13 and 14 are not promising catalysts for Li–S batteries, though for different reasons. Instead, metals like Ti, V, Nb, and Mo show enhanced LiPS binding (<−3 eV for Li_2_S adsorption), lower overpotential (less than 0.5 V using the vacuum model), and lower kinetic barriers (less than 1 eV) for activating the Li–S bonds. This group is followed by the second group of metals (Fe, Mn, Ru, and Cr), which have moderate binding (∼−2 eV for Li_2_S binding), moderate overpotential (0.5–0.8 V), and activation barriers (1.2–1.5 eV). Other metals like Co, Ni, Rh, and Pd show further compromised performance (weaker Li_2_S binding, slightly higher activation barriers, and overpotential than the second group) based on the discussion of the thermodynamics and kinetics. This prediction agrees with experimental studies in the literature, in which many of these metal centers (Fe, Co, V, Mo, Ni, etc.) have been tested with enhanced performance (improved capacity retention, high rate performance) in LSBs.^24,32^ Next, we experimentally investigate the performance of three representative elements—Nb, Fe, and Ni—based SACs to compare with our simulations.

Figure 5 presents the structural and compositional characterization of the atomically dispersed Nb SAC samples. The microstructure was examined by using SEM in both secondary electron (SE) and backscattered electron (BSE) modes. As depicted in Figure 5a and , the material exhibits a hierarchical porous structure composed of interconnected mesopores and micropores. This architecture is beneficial for accommodating sulfur species, enhancing the accessibility of active SAC sites, and promoting electrolyte infiltration. The observed porosity arises from the etching of the carbon matrix, primarily induced by the release of NH_3_ during the thermal decomposition of ammonium hydroxide chloride [(NH_3_OH)­Cl] in the pyrolysis process.

**Figure 5:**
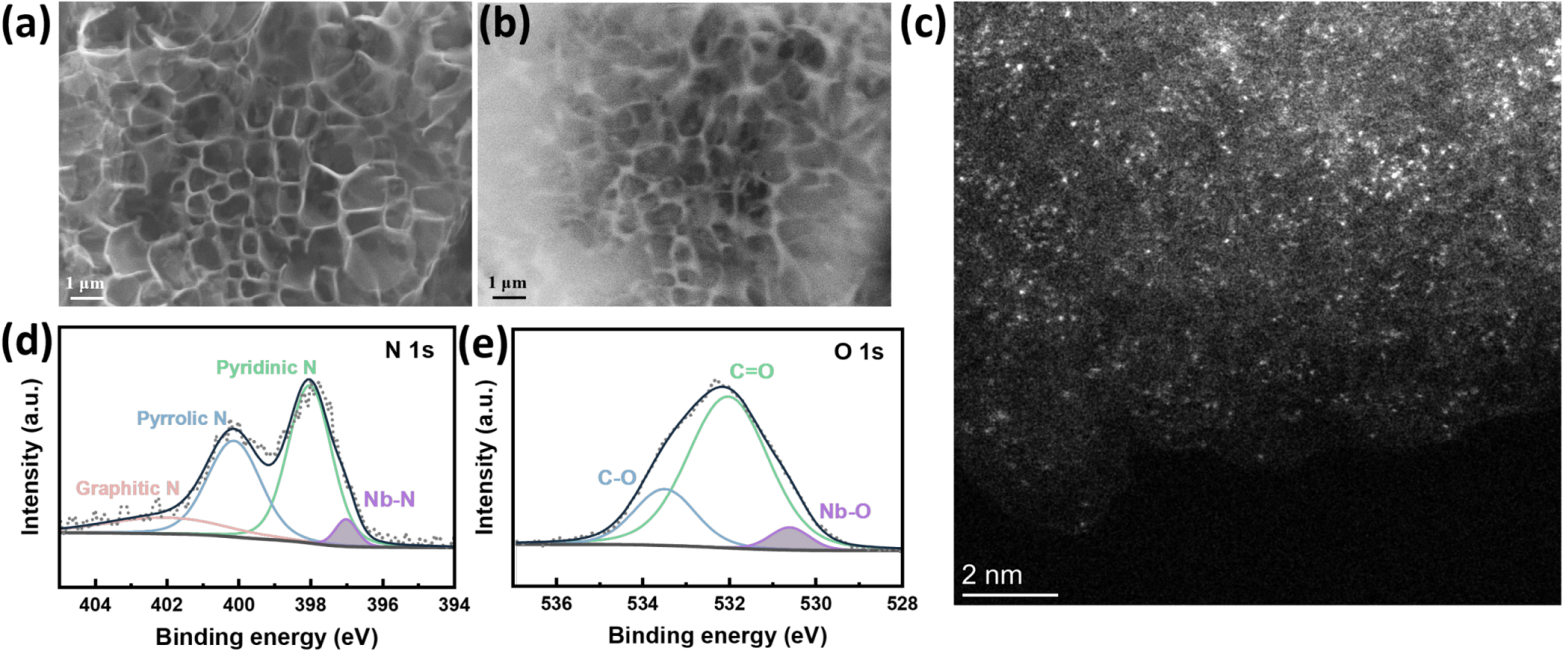
Structural characterization of Nb-SA/NC. (a) Secondary electron (SE) and (b) backscattered electron (BSE) SEM images of Nb-SA/NC. (c) AC-HAADF-STEM image of single Nb atoms (bright spots) from Nb-SA/NC. (d) N 1s and (e) O 1s XPS spectra of Nb-SA/NC.

To rule out the presence of metal nanoparticles, further microscopic imaging was performed by using the backscattered electron (BSE) mode. This technique allows for deeper electron penetration into the sample, providing enhanced material contrast based on the atomic number of the elements involved.^33^ BSE imaging is particularly useful for detecting both surface and subsurface features, making it ideal for the identification of buried nanoparticles or clusters. In the BSE images (Figure 5b, , and ), no bright spots indicative of Nb metallic nanoparticles were observed, suggesting their absence in the sample. Complementary energy-dispersive X-ray spectroscopy (EDS) analysis of the Nb-SA/NC samples (Figure S11c and f) confirms the presence of Nb, indicating that the element is atomically dispersed rather than forming larger aggregates.

Similarly, this diagnostic approach was applied to the synthesized Fe-SA/NC and Ni-SA/NC samples, as shown in Figures S12 and S13, respectively. These observations are further corroborated by the X-ray diffraction (XRD) patterns of Nb-SA/NC and other SAC samples (Figure S14), which display only two broad diffraction peaks at 26° and 45°, corresponding to the (002) and (101) planes of amorphous carbon.^34^ The absence of any characteristic peaks associated with Nb metal or other transition-metal species strongly supports the notion that no crystalline nanoparticles are present in these SAC materials.

The high-angle annular dark-field scanning transmission electron microscopy (HAADF-STEM) image in Figure 5c and the corresponding electron energy loss spectroscopy (EELS) in Figure S15 for Nb-SA/NC confirm the presence and distribution of Nb, N, O, and C, highlighting the carbon support doped with heteroatoms of N and O. The image in Figure 5c further visualizes the spatial distribution of Nb atoms. The numerous isolated bright spots observed in the images are characteristic of atomically dispersed Nb species anchored on the carbon matrix. Similarly, the formation of atomically dispersed Fe and Ni catalysts was verified through HAADF-STEM imaging, as shown in Figures S16 and S17, respectively. The accompanying EELS elemental mapping confirms the presence and uniform distribution of the respective metal atoms and heteroatoms, further supporting the successful synthesis of well-dispersed transition metal SACs.

During synthesis, the thorough dissolution and homogeneous dispersion of the Nb–metal precursor on the glucose-derived substrate played a critical role in preventing precipitation and achieving uniform distribution of metal centers prior to carbonization. Upon carbonization at elevated temperatures, the pyrolyzed metal precursors are transformed into atomically dispersed Nb sites. This method is notable for its simplicity and scalability, making it broadly applicable for the large-scale synthesis of single-atom catalysts (SACs). The resulting hierarchical porous carbon framework not only provides effective confinement for sulfur species but also incorporates heteroatom dopants (O and N) that, together with the dispersed Nb atoms, enhance the adsorption of lithium polysulfide intermediates and promote their redox conversion kinetics.^15^ This synergistic combination contributes to improved electrochemical performance by stabilizing sulfur species and accelerating polysulfide redox reactions.

BET analysis was used to probe the pore structures of the samples. As shown in Figure S18, the N_2_ adsorption/desorption isotherms reveal that Nb-SA/NC exhibits a high specific surface area of 581.96 m^2^ g^–1^ and a pore volume of 0.7639 cm^3^ g^–1^. Meanwhile, the other samples (Fe-SA/NC, Ni-SA/NC, and NC) showed comparable specific surface areas. The high surface area of Nb-SA/NC not only facilitates the uniform dispersion of metal atoms but also promotes lithium-ion diffusion and accommodates volume expansion during cycling. Moreover, the well-developed hierarchical porous structure provides exposed catalytic sites and contributes to the physical confinement of sulfur species.

X-ray photoelectron spectroscopy (XPS) was conducted to analyze the chemical composition and oxidation states of Nb-SA/NC. The wide-survey XPS spectrum (Figure S19) confirmed the presence of Nb, C, N, and O. The high-resolution N 1s spectrum (Figure 5d) can be deconvoluted into four distinct nitrogen species: pyridinic N at 398.1 eV, pyrrolic N at 400.1 eV, graphitic N at 401.9 eV, and Nb–N bonds at 397 eV,^35,36^ suggesting that nitrogen may serve as anchor sites for the Nb atoms. The Nb 3d spectrum of Nb-SA/NC (Figure S20a) exhibits two dominant peaks at 206.9 and 209.7 eV, corresponding to Nb^5+^ 3d_5/2_ and Nb^5+^ 3d_3/2_, respectively, indicating the +5 valence state of Nb.^37,38^ The C 1s spectrum (Figure S20b) reveals bonding configurations on the carbon substrate, with peaks at 284.8, 286.2, and 288.5 eV corresponding to C–C, C–(O, N), and C=O bonds, respectively.^39–41^ The O 1s spectrum (Figure 5e) displays peaks at 531.9 eV for C=O bonds and 533.3 eV for C–O bonds.^42,43^ Notably, the Nb–O peak is observed at 530.6 eV,^44^ suggesting that oxygen atoms may participate in the coordination environment of the central Nb atoms. Note that such detailed coordination was not included in the original DFT calculations that were targeted at screening different metals with the same atomic configuration. As discussed below, the experimentally measured battery charging/discharging performance over Nb-SA/NG in fact agrees with the general trend predicted by DFT calculations.

As shown in Figure S21, the oxidation states of Fe in Fe-SA/NC and Ni in Ni-SA/NC were revealed to be +3 and +2, respectively. In the high-resolution N 1s spectra, a distinct peak corresponding to the M–N bond was observed at 399.3 eV for Fe–N and 399.2 eV for Ni–N, which is characteristic of nitrogen atoms coordinated to transition metals.^45,46^ To validate the M–N_4_ configuration used in the DFT calculations, we performed quantitative XPS analysis by correlating the relative intensity of the M–N_*x*_ peak with the atomic percentage of the respective transition metal. For instance, the wide XPS survey of Nb-SA/NC (Figure S19) indicates a Nb atomic percentage of 0.11% and a total nitrogen atomic percentage of 8.27%. Deconvolution of the N 1s spectrum (Figure 5d) reveals that the Nb–N contribution accounts for 5.12% of the total N 1s peak area. Based on these values, the ratio of coordinated nitrogen atoms to Nb atoms on the surface was calculated as 3.85. This value is close to 4, consistent with the M–N_4_ moieties used in the computational studies. Similar analyses were conducted for Fe-SA/NC and Ni-SA/NC, providing coordination ratios of 3.75 and 3.81, respectively. A summary of these quantitative results is presented in Table S4.

To catalytically convert soluble LiPSs to solid Li_2_S_2_/Li_2_S, initial adsorption onto the catalyst surface is essential. We evaluated the polysulfide adsorption capability of different catalysts by immersing 50 mg of catalyst powder in 10 mL of 0.005 M Li_2_S_6_ solution for 6 h and observing the resulting color changes. As shown in Figure S22, the initially dark yellow Li_2_S_6_ solution became nearly colorless after treatment with Nb-SA/NC, while Fe-SA/NC, Ni-SA/NC, and pristine nitrogen-doped carbon (NC) exhibited progressively weaker decoloration. The hierarchical porous structure of the carbon support contributed to physical adsorption, while the presence of metal centers significantly enhanced chemical interactions with LiPSs. Among them, Fe showed stronger adsorption than Ni, but only Nb-SA/NC completely decolorized the solution, indicating the strongest adsorption capability—consistent with theoretical predictions. This trend was further confirmed by UV–vis spectroscopy of the Li_2_S_6_ electrolyte postadsorption^47,48^ (Figure S22), where Nb-SA/NC exhibited the greatest decrease in absorbance intensity. The overall adsorption efficiency followed the order: Nb-SA/NC > Fe-SA/NC > Ni-SA/NC > NC, aligning with both visual observations and predicted interactions between LiPSs and the single-atom metal sites.

To assess the catalytic performance of Nb-SA/NC in Li–S redox reactions, a series of electrochemical measurements were conducted. Figure 6a compares the impact of SACs on cyclic voltammetry (CV) profiles of Li–S full cells recorded at a scan rate of 0.1 mV s^–1^. The CV curves clearly show two cathodic peaks: Peak I, corresponding to the conversion of S_8_ to soluble Li_2_S_*n*_ (6 ≤ *n* ≤ 8), and Peak II, corresponding to the subsequent reduction to Li_2_S. An oxidation peak at approximately 2.3 V is attributed to the reverse oxidation reactions, where sulfur is produced from Li_2_S and Li_2_S_*n*_.^49^ Notably, Nb-SA/NC-based LSBs exhibited the highest reduction potential and the lowest oxidation potential among tested catalysts, followed by Fe-SA/NC, Ni-SA/NC, and the bare NC counterpart. This trend indicates reduced polarization and enhanced redox kinetics. Furthermore, the sharpest redox peaks and highest peak current densities observed for Nb-SA/NC, relative to NC, provide compelling evidence of its superior electrocatalytic activity in promoting the conversion of soluble LiPS intermediates.

**Figure 6:**
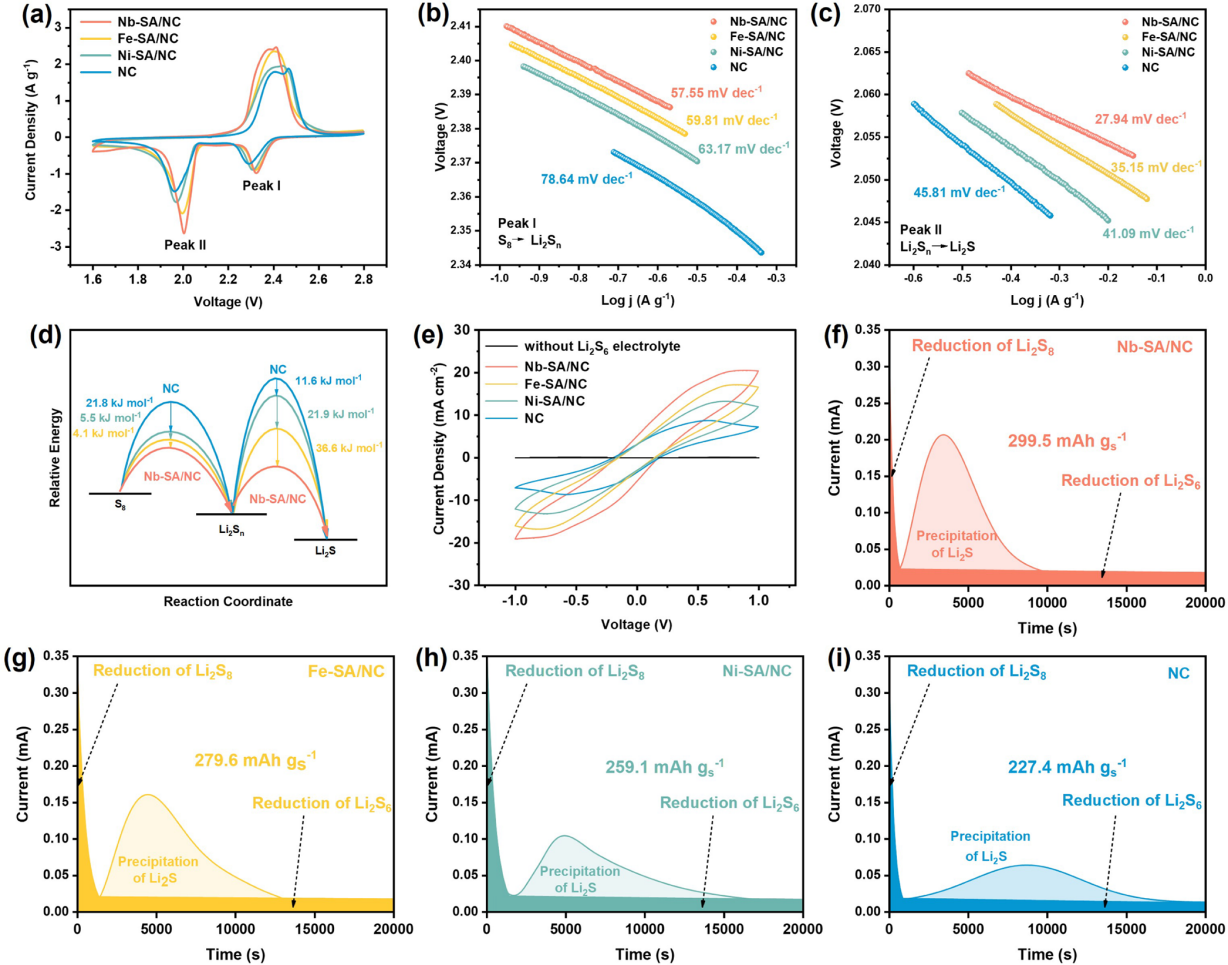
Catalytic behavior evaluation of different catalysts. (a) CV profiles of Li–S full cells assembled with sulfur cathodes incorporating Nb-SA/NC, Fe-SA/NC, Ni-SA/NC, and NC at a scan rate of 0.1 mV s^–1^. Corresponding Tafel plots derived from discharge Peak I (b) and Peak II (c). (d) Activation energies for sulfur reductions. (e) CV profiles of various Li_2_S_6_ symmetric cells at a 50 mV s^–1^ scan rate. Potentiostatic Li_2_S precipitation curves acquired on (f) Nb-SA/NC, (g) Fe-SA/NC, (h) Ni-SA/NC, and (i) NC active electrode surfaces.

Tafel slopes were calculated from the CV profiles at Peaks I and II to quantitatively evaluate the catalytic activity during the sulfur reduction process. As shown in Figure 6b, the Tafel slopes at Peak I for sulfur cathodes with Nb-SA/NC, Fe-SA/NC, Ni-SA/NC, and NC were 57.55, 59.81, 63.17, and 78.64 mV dec^–1^, respectively. For Peak II, the corresponding slopes were 27.94, 35.15, 41.09, and 45.81 mV dec^–1^ (Figure 6c). The consistently lowest Tafel slopes observed for Nb-SA/NC indicate the fastest reaction kinetics during both stages of sulfur reduction, confirming its superior electrocatalytic activity in facilitating LiPS conversion.

The relative activation energy (*E*_a_) for the discharge process was then derived from the Tafel plots using the equation given as $E_{a}=E_{a}^{0}-\frac{RT}{b}\varphi_{\mathrm{Red},\mathrm{ir}}$ , where *b* is the Tafel slopes, and φ_Red,ir_ represents the irreversible potential obtained from CV curves. As shown in Figure 6d, Nb-SA/NC exhibits significantly reduced activation energies—by 31.2 and 69.8 kJ mol^–1^—for the stepwise sulfur reduction reactions from S_8_ to Li_2_S_n_ and Li_2_S_*n*_ to Li_2_S, respectively. Specifically, for the S_8_ → Li_2_S_*n*_ transition, Nb-SA/NC showed an *E*_a_ that was 4.1 kJ mol^–1^ lower than Fe-SA/NC, Fe was 5.5 kJ mol^–1^ lower than Ni-SA/NC, and Ni was 21.8 kJ mol^–1^ lower than pristine NC. For the Li_2_S_*n*_ → Li_2_S conversion, Nb’s *E*_a_ was 36.6 kJ mol^–1^ lower than Fe, Fe was 21.9 kJ mol^–1^ lower than Ni, and Ni was 11.6 kJ mol^–1^ lower than NC. These reductions in activation energy confirm the enhanced catalytic effect of Nb in accelerating LiPS redox conversion by lowering the energy barrier.^50,51^ This trend aligns well with our DFT calculations of the kinetic barriers for Li–S bond activation (Figure 4). However, it is worth noting that the DFT simulations did not account for electrode potential or solvent effects, making direct quantitative comparisons challenging.

Symmetric cells were assembled by using identical SAC-incorporated electrodes and a Li_2_S_6_-containing electrolyte to investigate the redox kinetics of LiPSs. As shown in Figure 6e, the symmetric CV curve of the Li_2_S_6_-free electrolyte displays a negligible capacitive current, confirming the absence of redox activity. In contrast, electrodes loaded with Nb-SA/NC exhibit significantly higher and more reversible redox currents compared to those with NC, Fe-SA/NC, and Ni-SA/NC. The observed current densities follow the order: Nb > Fe > Ni > NC, indicating enhanced catalytic activity for LiPS conversion facilitated by the Nb single-atom sites. This trend is consistent with the theoretical predictions of increased activity for Nb-based catalysts. Additionally, electrochemical impedance spectroscopy (EIS) results from freshly assembled cells (Figure S23) show that the Nb-SA/NC-based symmetric cell possesses the lowest charge transfer resistance. This suggests that the Nb–N coordination environment effectively promotes interfacial charge transfer and accelerates the redox kinetics of LiPSs, further supporting the superior catalytic role of Nb in polysulfide conversion.

Finally, to investigate the influence of Nb SACs on enhancing liquid–solid phase conversions of LiPSs, potentiostatic Li_2_S precipitation tests were conducted using cells with a Li_2_S_8_ catholyte. The resulting nucleation curves, shown in Figure 6f–i, illustrate the phase transformation behavior on different catalyst-based electrode substrates. Initially, the current decreases due to nonfaradaic double-layer charging and the reduction of high-order polysulfides (e.g., Li_2_S_8_). This is followed by a rise in current, reaching a peak that corresponds to the nucleation of solid Li_2_S, and then a gradual decline as the insulating Li_2_S layer forms, eventually halting the reaction.^52,53^ Among the tested electrodes, Nb-SA/NC demonstrated the highest Li_2_S deposition capacity of 299.5 mA h g^–1^, outperforming Fe-SA/NC (279.6 mA h g^–1^), Ni-SA/NC (259.1 mA h g^–1^), and bare NC (227.4 mA h g^–1^). In addition, the earlier onset and higher peak current observed for Nb-SA/NC indicate a faster nucleation process and more efficient Li_2_S growth. These results confirm that Nb SACs significantly reduce the nucleation energy barrier and enhance the kinetics of Li_2_S precipitation, thereby promoting faster and more complete liquid-to-solid phase transitions during discharge.

SEM characterization after Li_2_S precipitation tests revealed distinct deposition morphologies, depending on the effect of different metal centers in the SACs. As shown in Figure S24a, Nb-SA/NC with abundant nucleation sites promotes the formation of uniform, nanosized spherical Li_2_S clusters. In the case of Fe-SA/NC (Figure S24b), Li_2_S still adopts a particle-like structure; however, the deposition is less uniform, with a tendency to agglomerate into larger clusters. In contrast, Li_2_S deposited on Ni-SA/NC (Figure S24c) exhibits an elongated morphology, resulting in lateral or rod-like structures. Without the mediation of single-atom active centers, as in the pristine NC (Figure S24d), Li_2_S nucleation is sparse and uncontrolled, resulting in the formation of irregular, bulky deposits that lead to a passivating film covering the electrode surface.^54^

For the Li_2_S dissolution tests, the assembled cells were first galvanostatically discharged to 1.7 V at a current of 0.112 mA, followed by a potentiostatic charge at 2.4 V until the current decreased below 0.01 mA.^55^ As shown in Figure S25, the initial rising current and subsequent current peak are determined by the reaction rate, which is dominated by kinetic factors such as the electronic conductivity of Li_2_S, the diffusivity of Li^+^ within Li_2_S, and the charge transfer at the Li_2_S surface.^56^ Notably, the Nb-SA/NC cathode showed the highest and earliest charging current peak among all of the samples, resulting in the largest Li_2_S dissolution capacity of 509.2 mA h g^–1^. The superior performance of Nb-SA/NC can be attributed to its lower activation energy for Li–S bond dissociation, as revealed by DFT calculations, and its rapid charge transfer at the interface between Nb active sites and Li_2_S as well as rich electron transport paths inside the downsized and uniform Li_2_S, which collectively accelerate the dissociation process.

After the Li_2_S dissolution tests, SEM characterization showed distinct differences in the surface morphology across different active metal centers. As shown in Figure S26a, the Nb-SA/NC electrode surface appears clean, suggesting that almost all Li_2_S has been oxidized, which demonstrates a reversible solid–liquid conversion facilitated by the Nb active center. By comparison, a few spherical Li_2_S clusters remained on Fe-SA/NC (Figure S26b), while a small number of unreacted residual Li_2_S islands are observed on the surface of the Ni-SA/NC (Figure S26c). In contrast, a large area of porous Li_2_S films is left on the surface of the pristine NC (Figure S26d), implying that a large portion of Li_2_S is not electrochemically utilized during the oxidation reaction and has difficulty participating in dissociation without the presence of single-atom metal active centers. This is consistent with the dissolution tests.

Rate capability is a critical parameter for evaluating the performance of lithium–sulfur batteries. As shown in Figure 7a, Li–S full cells assembled with Nb-SA/NC-based sulfur cathodes delivered high discharge capacities of 1418.9, 1167.2, 966.6, 892.8, 817.5, and 679.3 mA h g^–1^ at current rates of 0.1, 0.3, 0.5, 1, 3, and 5 C, respectively—substantially outperforming the NC-based reference cathodes. Notably, when the current rate was returned to 3 C, a capacity of 819.8 mA h g^–1^ was recovered, indicating excellent kinetic reversibility and structural stability, attributed to the catalytic effect of Nb single-atom sites. The galvanostatic charge–discharge profiles of Nb-SA/NC-based LSBs at a high rate of 5 C (Figure 7b) show well-defined two-step discharge plateaus, characteristic of efficient sulfur redox reactions. In comparison, LSBs incorporating Fe-SA/NC and Ni-SA/NC cathodes delivered lower capacities of 604.6 and 510.3 mA h g^–1^, respectively, along with increased voltage polarization. Meanwhile, the NC-based sulfur cathode exhibited a pronounced voltage gap and severely diminished discharge plateau at 5 C (Figure S27), highlighting the inferior rate performance in the absence of catalytic metal centers.

**Figure 7:**
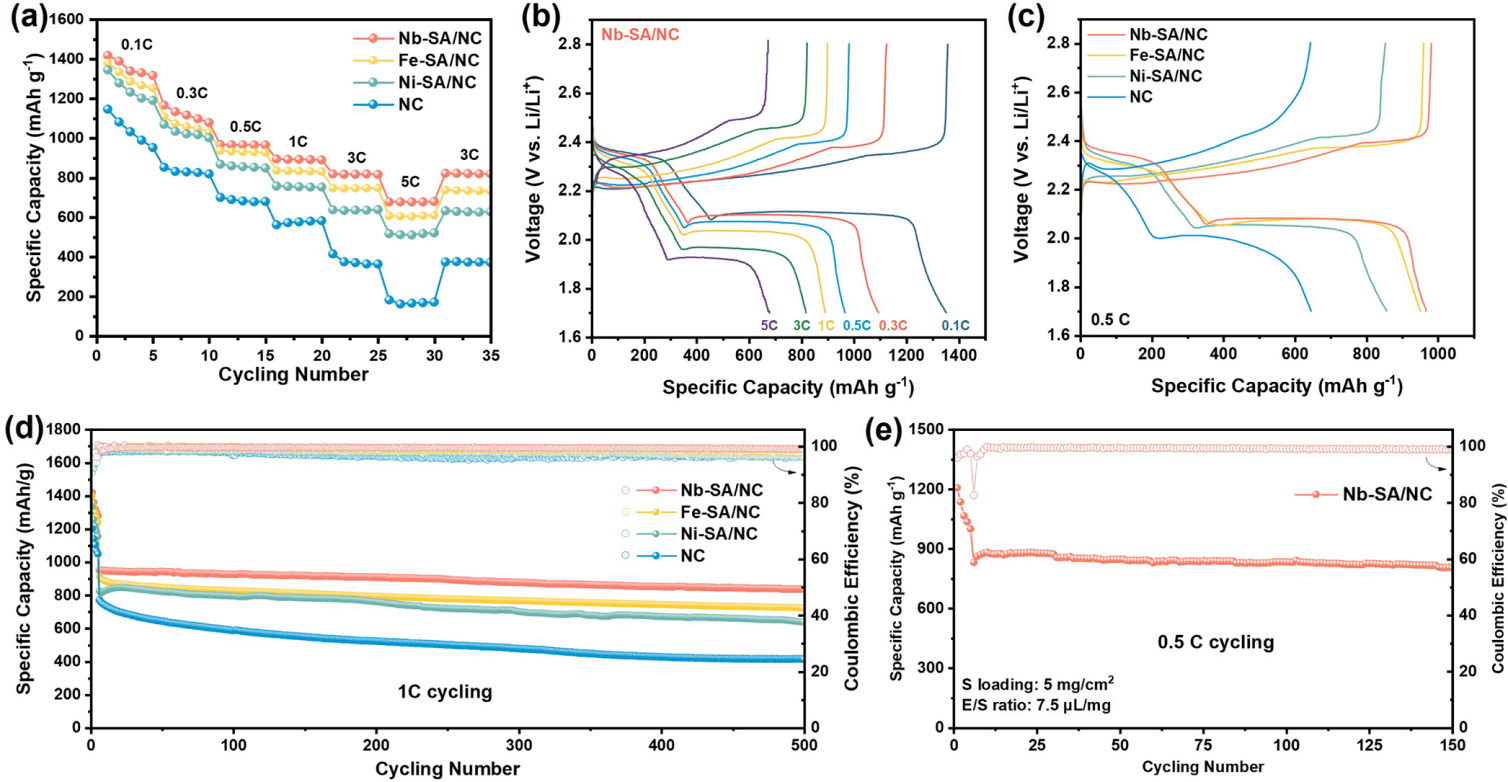
Electrochemical performance of Li–S batteries assembled with different catalyst-based sulfur cathodes. (a) Rate performance of Li–S full cells at various current densities using sulfur cathodes incorporating Nb-SA/NC, Fe-SA/NC, Ni-SA/NC, and NC. (b) Galvanostatic charge–discharge voltage profiles of the Nb-SA/NC-based Li–S cell measured from 0.1 C to 5 C. (c) Comparison of voltage polarization at 0.5 C among Li–S cells with Nb-SA/NC, Fe-SA/NC, Ni-SA/NC, and NC cathodes. (d) Long-term cycling performance of Li–S cells at 1 C for different catalyst systems. (e) Cycling stability of Nb-SA/NC-based Li–S cells under practical conditions with a high sulfur loading of 5 mg cm^–2^ and a low electrolyte-to-sulfur (E/S) ratio of 7.5 μL mg^–1^.

As shown in Figure S28a, Nb-SA/NC exhibits a lower positive overpotential during the initial charging stage, indicating a reduced energy barrier for the decomposition of solid Li_2_S into soluble polysulfides. This behavior can be attributed to the strong Nb–Li_2_S interaction, as supported by the DFT calculations. Similarly, the less negative overpotential observed at the second discharge plateau (Figure S28b) suggests a lower activation barrier for the reverse conversion from soluble LiPSs to solid Li_2_S, consistent with the results from the Li_2_S precipitation tests. The overall reduction in overpotentials during both charge and discharge processes, in agreement with DFT-predicted values (Figure 3), highlights the dual-function catalytic capability of Nb SACs in facilitating both Li_2_S decomposition and formation. Consequently, as illustrated in Figure 7c, LSBs assembled with Nb-SA/NC exhibit the lowest charge–discharge polarization among all tested samples at the same current rate.

Long-term cycling stability with high-capacity retention was also achieved in LSBs incorporating Nb-SA/NC, as shown in Figure 7d. After 500 cycles at 1 C, the Nb-SA/NC-based LSB maintained a discharge capacity of 837.5 mA h g^–1^ with a high Coulombic efficiency (CE) exceeding 98.6%, corresponding to an average capacity decay rate of just 0.023% per cycle. In comparison, cells assembled with Fe-SA/NC and Ni-SA/NC stabilized at 726.6 and 639.4 mA h g^–1^, with CEs of 97.95% and 97.36% and decay rates of 0.041% and 0.046% per cycle, respectively. By contrast, the NC-based reference cathode showed rapid capacity fading, decreasing to 493.2 mA h g^–1^ after 500 cycles with a decay rate of 0.072% per cycle. The poor performance of the NC-based system highlights the weaker interaction with LiPSs, which leads to their diffusion into the electrolyte and subsequent loss of active material. The superior stability and efficiency of Nb-SA/NC are attributed to its strong adsorption of LiPSs/Li_2_S and its ability to reduce the overpotential and activation energy barriers for LiPS redox conversion. These observations are consistent with theoretical predictions and corroborated by a series of electrochemical measurements.

For practical applications of LSBs, a higher sulfur loading with a lower electrolyte-to-sulfur (E/S) ratio is desired. Nb-SA/NC-based LSBs with a high sulfur loading of 5 mg cm^–2^ and an E/S ratio of 7.5 μL mg^–1^ were tested. Prior to cycling, the assembled cell was first activated at 0.1 C for 5 cycles to ensure sufficient electrolyte wetting. As shown in Figure 7e, the cell incorporated with Nb-SA/NC retained a capacity of 806.5 mA h g^–1^, or 4 mA h cm^–2^ after cycling at 0.5 C for 150 cycles.

In contrast to the more straightforward dissolution–carbonization process at 600 °C employed in this work, a recent study^28^ reported Nb SACs synthesized via high-temperature carbonization at 960 °C, achieving remarkable LSB performance with over 85% capacity retention after 1000 cycles and a high-rate capacity of 740 mA h g^–1^ at 7 C. These results further underscore the strong catalytic potential of Nb SACs in promoting sulfur redox reactions and their promise for the development of practical lithium–sulfur batteries.

## Conclusion

Through a combined study of computation and experiments, we report that a group of metals (Ti, V, Mo, and Nb) incorporated into graphitic carbon has promising catalytic properties due to three factors—strong binding energy of lithium sulfides, reduced overpotential of the redox chemistry, and low kinetic barrier for the Li–S bond activation. Our computational analysis indicates that metal centers can be categorized into three groups—represented by Nb, Fe, and Ni—each exhibiting different levels of catalytic capability toward sulfur redox reactions. Guided by theoretical predictions, we synthesized atomically dispersed Nb, Fe, and Ni SACs and systematically evaluated their catalytic performance. Nb-SA/NC exhibited the strongest LiPS adsorption, lowest activation energy for Li_2_S formation, and the fastest redox kinetics among all samples. As a result, LSBs with Nb-SA/NC delivered a high-rate capacity of 679.3 mA h g^–1^ at 5 C and retained 837.5 mA h g^–1^ after 500 cycles at 1 C with only 0.023% capacity decay per cycle. This work presents an effective strategy that combines theoretical screening and experimental validation in exploring SACs for LSBs.

## Computational and Experimental Methods

### Computational Details

The plane-wave density functional theory as implemented in the Vienna Ab-initio Simulation Package (VASP)^57^ was used in the system to calculate energy and electronic structure. The projector augmented wave (PAW) formalism^58^ of the Perdew–Burke–Ernzerhof (PBE) functional^59^ within the generalized gradient approximations (GGA) was used to describe the exchange-correlation energy. The plane-wave cutoff energy was set to 400 eV. The Gaussian smearing method was used, and the width of smearing was chosen as 0.02 eV. For the binding energy and adsorption conformation simulations, we used the DFT-D3 approach^60^ to include the van der Waals interaction. The Brillouin zone was sampled using Monkhorst–Pack scheme with a k-point mesh of 3 × 3 × 1 in the Γ-centered grids for the structural relaxation.^61^ The structure relaxation was continued until the forces on all the atoms converged to less than 0.02 eV/Å and the total energy change between two steps was smaller than 10^–5^ eV. The activation barrier (*E*_a_) for Li_2_S decomposition was determined by calculating the transition states using the climbing image nudged elastic band (CI-NEB)^62^ and the dimer methods,^63^ verified by a single imaginary vibrational frequency along the reaction coordinates.

DFT calculations with semilocal functions have the challenge of describing the on-site Coulomb interaction of localized electrons, which may influence calculation results.^64^ To investigate this possibility, we tested calculations by using the DFT+U method for V-SA/NC, Co-SA/NC, Pd-SA/NC, and Li_2_S on these surfaces. The appropriate U values reported in the literature depend on the fitted properties, such as the band gap, the Δ*H* of formation of the oxide, and the lattice parameter, which are usually in the range of 3 to 5 eV.^65^ In these test calculations, *U* = 3, 3.5, 4, 4.5, and 5 eV were used. As shown in Table S3, the charge distributions on V-SA, Co-SA, and Pd-SA calculated by DFT+U are slightly higher than those obtained using DFT PBE, but the overall trend remains consistent. Furthermore, the adsorption trend of V-SA, Co-SA, and Pd-SA/NC with Li_2_S also aligns, as illustrated in Figure S29. Since our focus is on qualitative trend analysis and there are no experimental adsorption values for reference to benchmark the *U* value for the SACs, PBE functionals were used for subsequent calculations without including the U correction. It is also anticipated that, as the thermodynamics and kinetics in the charging and discharging processes are determined by the difference between Li_2_S*, LiS*, and S*, as shown in Figures 3 and 4, the effect of the *U* values on adsorption energy will likely be canceled out.

The periodic structural model includes 154 carbon atoms with a divacancy created in the middle. Four nitrogen atoms were incorporated around the divacancy to coordinate with the metal atoms. In the vertical direction, a vacuum layer of about 20 Å was introduced for all the surfaces. To compare the intrinsic properties of metals and their impact on the LiPS redox chemistry, the same basic model, that is, the same bonding configuration of the metal centers (i.e., metal-N_4_ embedded in graphene) was applied here to all the metal centers, each of which was then fully optimized with and without adsorbates. The crystal orbital Hamilton population (COHP)^66^ was calculated using the LOBSTER program.^67^

Different from reduction steps (e.g., from Li_2_S_4_, Li_2_S_2_ to Li_2_S) investigated in many previous works,^1,10^ here we investigated the oxidation reactions (from Li_2_S to LiS and then to S), which are more relevant to the charging process of the battery. The fundamental insights should be similar between these two approaches as both involve the activation of the Li–S bond. The overpotential of two-electron transfer was calculated as follows. The two steps are depicted in eqs 1 and 2.

1 $${\mathrm{Li}}_{2}S^{*}\to {\mathrm{LiS}}^{*}+{\mathrm{Li}}^{+}+e^{-}$$

2 $${\mathrm{LiS}}^{*}\to S^{*}+{\mathrm{Li}}^{+}+e^{-}$$

According to eqs 1 and 2, the Gibbs free energy of the two steps can be written as

3 $$\Delta G_{1}=G\left({\mathrm{LiS}}^{*}\right)+G\left(\mathrm{Li}\right)-G\left({\mathrm{Li}}_{2}S^{*}\right)$$

4 $$\Delta G_{2}=G\left(S^{*}\right)+G\left(\mathrm{Li}\right)-G\left({\mathrm{LiS}}^{*}\right)$$

In addition to examining the reaction under vacuum conditions, we also explored the reaction taking place in an electrolyte solvent using a microsolvation model. In this approach, we consider the interaction of each Li^+^ ion in Li_2_S and LiS with a 1,3-dioxolane (DOL) molecule (Figure S1), along with their interaction with active sites. The following equations include the DOL solvents, similar to previous works in the literature:^10^

5 $$2{\mathrm{DOL‐Li}}_{2}S^{*}\to {\mathrm{DOL‐LiS}}^{*}+\mathrm{DOL}+{\mathrm{Li}}^{+}+e^{-}$$

6 $${\mathrm{DOL‐LiS}}^{*}\to S^{*}+\mathrm{DOL}+{\mathrm{Li}}^{+}+e^{-}$$

In which 2DOL-Li_2_S* represents adsorbed Li_2_S solvated by two DOL molecules, and DOL-LiS* represents adsorbed LiS solvated by one DOL molecule; they are illustrated in Figure 3.

This investigation enabled us to assess how the solvent affects the observed catalytic performance trends across various substrates. The free energy of reactions in the presence of a solvent was calculated as follows:

7 $$\Delta G_{1}^{\prime }=G\left(1{\mathrm{DOL‐LiS}}^{*}\right)+G\left(\mathrm{Li}\right)+G\left(\mathrm{DOL}\right)-G\left(2{\mathrm{DOL‐Li}}_{2}S^{*}\right)$$

8 $$\Delta G_{2}^{\prime }=G\left(S^{*}\right)+G\left(\mathrm{Li}\right)+G\left(\mathrm{DOL}\right)-G\left({\mathrm{DOL‐LiS}}^{*}\right)$$

where the *G*(1DOL-LiS*) stands for the free energy of the adsorbed LiS complex with one DOL molecule, and *G*(DOL) denotes the free energy of a DOL molecule in implicit solvation. In eqs 7 and 8, *G*(Li^+^) + *G*(e^–^) are written in the form of *G*(Li), following a similar approach as the computational hydrogen electrode model:^68^

9 $$G\left({\mathrm{Li}}^{+}\right)+G\left(e^{-}\right)=G\left(\mathrm{Li}\right)-eU$$

The *G*(Li) represents the Li solid state, while *U* refers to the potential versus the Li/Li^+^ electrode. In this study, *G*(Li^+^) + *G*(e^–^) was considered at 0 V versus the Li/Li^+^ electrode.^10^ The vibrational contribution to the Gibbs free energy for all solid-state species was included as follows:

10 $$G=E+\mathrm{ZPE}-TS_{\mathrm{vib}}$$

Peng et al.^10^ reported that for the adsorption of lithium–sulfur species, the correction from *E* to *G* (ZPE – *TS*_vib_) is approximately 0. Therefore, in the following, we also assumed that the Gibbs free energy for lithium–sulfur species is equal to their DFT-calculated electronic energy. The step has the highest reaction energy (Δ*G*_Max_) is considered the potential-determining step, which is compared between the values of Δ*G*_1_ and Δ*G*_2_. The overpotential can be calculated as

11 $$\eta =\frac{\Delta G_{\mathrm{Max}}}{e}-E_{0}$$

where *E*_0_ is the standard reduction potential, calculated based on the overall reaction of the Li–S battery:

12 $$E_{0}=\left(2G\left(\mathrm{Li}\right)+G\left(S\right)-G\left({\mathrm{Li}}_{2}S\right)\right)/2e=2.17\;V$$

This calculated value is similar to what we calculated in the past using sulfur and lithium bulk as the reference.^15^

## Experimental Studies

### Preparation of M-SA/NC

The niobium SAC anchored on an N-doped carbon support (Nb-SA/NC) was synthesized through a controlled process involving dissolution, drying, and carbonization. Initially, 288 mg of glucose (C_6_H_12_O_6_) was dissolved in 80 mL of ethanol. Simultaneously, 11.5 mg of niobium­(V) chloride (NbCl_5_) was utilized as the metal salt precursor, which was ultrasonically dissolved with 1.38 g of hydroxylamine hydrochloride ((NH_3_OH)­Cl) in 80 mL of deionized water. The ethanol solution containing glucose and the aqueous solution containing (NH_3_OH)Cl and NbCl_5_ were mixed together. The obtained mixture was dried in a drying oven at a temperature of 70 °C for a duration of 12 h to remove any solvent and facilitate the formation of a stable precursor material. The dried mixture was then transferred to a crucible and subjected to a pyrolysis process. The temperature was raised gradually from room temperature to 600 °C, with a ramp rate of 5 °C min^–1^ and maintained at 600 °C for 4 h under an Ar atmosphere to complete the carbonization process. The obtained catalyst powder was then acid-leached in 2 M HCl at 80 °C for 2 h, followed by thorough rinsing with DI water and drying for further use. Similarly, Fe-SA/NC and Ni-SA/NC were synthesized by substituting NbCl_5_ with 21.2 mg of C_12_H_22_O_14_Fe and 12.6 mg of Ni­(NO_3_)_2_·6H_2_O, respectively, following the same protocol.^69^ The pristine nitrogen-doped carbon substrate (NC) used as a reference was prepared using the same procedure without the addition of metal salts.

### Materials Characterization

The porous morphologies and the energy-dispersive X-ray spectra (EDS) of the prepared samples were characterized by scanning electron microscopy at 30 kV (SEM, JEOL JXA-8530F) in secondary electron (SE) and backscattered electron (BSE) modes. The crystalline structure was analyzed by X-ray diffraction (XRD) with Cu Kα radiation (λ = 0.1541 nm) on a Malvern PANalytical Aeris diffractometer. X-ray photoelectron spectroscopy (XPS) measurements were performed on a Kratos Axis Supra+ spectrometer using Al Kα (1486.6 eV) as the excitation source. A charge neutralizer was used during measurements to minimize differential charging, and all spectra were calibrated against the C 1s peak at 284.8 eV. Peak fitting was performed consistently by using a Shirley background and constrained Gaussian–Lorentzian line shapes. Aberration-corrected high-angle annular dark-field scanning transmission electron microscopy (AC-HAADF-STEM) and elemental mapping were recorded on Themis microscopy at the University of Connecticut. A Dcorr+ spherical probe corrector is equipped with this microscope, giving the best spatial resolution of 0.08 nm. SuperX G1 was used for highly efficient STEM-EDX elemental mapping with a 0.7 sr EDX collection angle. The UV–vis absorbance spectroscopy after polysulfide adsorption tests was acquired by the Perkin Lambda 950 UV–Vis spectrometer.

### Fabrication of Nb-SA/NC-Based Sulfur Electrodes and Electrochemical Measurements

The sulfur cathode was prepared by a conventional slurry coating method. The carbon/sulfur composite was prepared by grinding the catalysts with sublimed sulfur into fine powders in a mass ratio of 1:4, followed by a melt-diffusion process at 155 °C for 12 h. The slurry was prepared by mixing the obtained composite (70 wt %), carbon black (20 wt %), and PVDF (10 wt %) in *N*-methyl-2-pyrrolidinone (NMP) solvent. Then, the slurry was coated onto carbon-coated aluminum foil by doctor-blade casting. The sulfur cathode was punched into a 1.13 cm^2^ disk with a sulfur loading of ∼1.5 or 5 mg cm^–2^. For comparison purposes, NC-based sulfur electrodes were also prepared by a similar procedure without SACs. CR2016-type Li–S coin cells were assembled in an argon-filled glovebox using the prepared SACs with S composite cathodes, Celgard 2400 membranes as separators, and polished lithium chips as anodes. The ether-based electrolyte was prepared by dissolving 1.0 M lithium bis­(trifluoromethanesulfonyl)­imide (LiTFSI) in a 1:1 (v/v) mixture of 1,2-dimethoxyethane (DME) and 1,3-dioxolane (DOL), with 2 wt % lithium nitrates (LiNO_3_) as an additive. The electrolyte-to-sulfur (E/S) ratio for typical electrochemical tests was 15 μL/mg, while a lower E/S ratio of 7.5 μL/mg was used in the 0.5 C cyclability test of a high sulfur loading of 5 mg cm^–2^.

The cyclic voltammograms (CV) of the assembled Li–S full cells were recorded over a voltage range of 1.6 to 2.8 V at a scan rate of 0.1 mV s^–1^. Electrochemical impedance spectroscopy (EIS) was performed in the frequency range of 100 kHz to 0.01 Hz with an AC voltage amplitude of 5 mV. Both CV and EIS measurements were conducted using a Biologic SP-240 electrochemical workstation. The galvanostatic charge–discharge curves of the Li–S cells were acquired using a LANDCT2001A battery tester at various current rates within a voltage range of 1.7 to 2.8 V.

Li_2_S_6_ symmetric cells and CV measurement: Active electrodes consisting of 90 wt % active materials (either M-SA/NC or NC) and 10 wt % PVDF were fabricated using the slurry-casting method on carbon-coated aluminum foil and were employed in Li_2_S_6_ symmetric cell CV tests and Li_2_S precipitation tests. A 0.25 M Li_2_S_6_ solution was prepared by mixing Li_2_S and sulfur in a 1:5 molar ratio in the blank electrolyte and stirring at 60 °C until the sulfur was fully dissolved. Li_2_S_6_ symmetric cells were assembled using identical active electrodes, each loaded with 20 μL of 0.25 M Li_2_S_6_ electrolyte, serving as both working and counter electrodes. The CV measurements of the Li_2_S_6_ symmetric cells were conducted at a scan rate of 50 mV s^–1^ within a potential window of −1 to 1 V to evaluate the polysulfide conversion kinetics.

Li_2_S precipitation tests: A 0.25 M Li_2_S_8_ solution was prepared by mixing Li_2_S and sulfur in a 1:7 molar ratio in a blank electrolyte. Then, 20 μL of the 0.25 M Li_2_S_8_ catholyte was dropped onto the active electrodes, and an additional 20 μL of the blank electrolyte was added to the Li anode. The assembled cell was first galvanostatically discharged at a constant current of 0.112 mA to 2.12 V and then held potentiostatically at 2.11 V until the current decreased to 1 × 10^– 5^ mA.^52^

## Supplementary Material





## Notes

Atomics structures of metal SA/NC; additional projected density of states and COHP analysis; table of the calculated reaction energies; SEM and XRD analysis of the SAC sample; HAADF–STEM images; additional XPS analysis; EIS Nyquist plots; results from Li_2_S_6_ symmetric cells; additional charging/discharging comparison between different SACs (PDF)
(ZIP)
