# Supplementary material for: Rational Design of Metal-Doped Graphitic Materials for Enhanced Lithium–Sulfur Batteries
Source: ACS Nano. 2025 Oct 14;19(42):37013–26. doi: 10.1021/acsnano.5c10546 (PMC12574205; doi:10.1021/acsnano.5c10546)
Supplement: Supplementary file 1 [file nn5c10546_si_001.pdf]

Supplementary Materials

Rational Design of Metal-Doped Graphitic Materials for Enhanced Lithium Sulfur Batteries

Vy Nguyen[1a], Xueyan Lin[2a], Rishav Baranwal[2], Haiyan Tan[3], David Wright[4], Zhaoyang Fan[5*], Bin Wang[1,6*]

[1] School of Sustainable Chemical, Biological and Materials Engineering, University of Oklahoma, Norman, OK 73019, USA

[2] School for Engineering of Matter, Transport & Energy, Arizona State University, Tempe, AZ 85281, USA

[3] Institute of Materials Science, University of Connecticut, Storrs, CT 06269, USA

[4]LeRoy Eyring Center for Solid State Science, Arizona State University, Tempe, AZ, 85287, USA

[5] School of Electrical, Computer and Energy Engineering, Arizona State University, Tempe, AZ 85281, USA

[6] Max Planck Institute for Sustainable Materials GmbH, D–40237 Düsseldorf, Germany

a. These authors contributed equally

Email: zyfan@asu.edu; wang_cbme@ou.edu

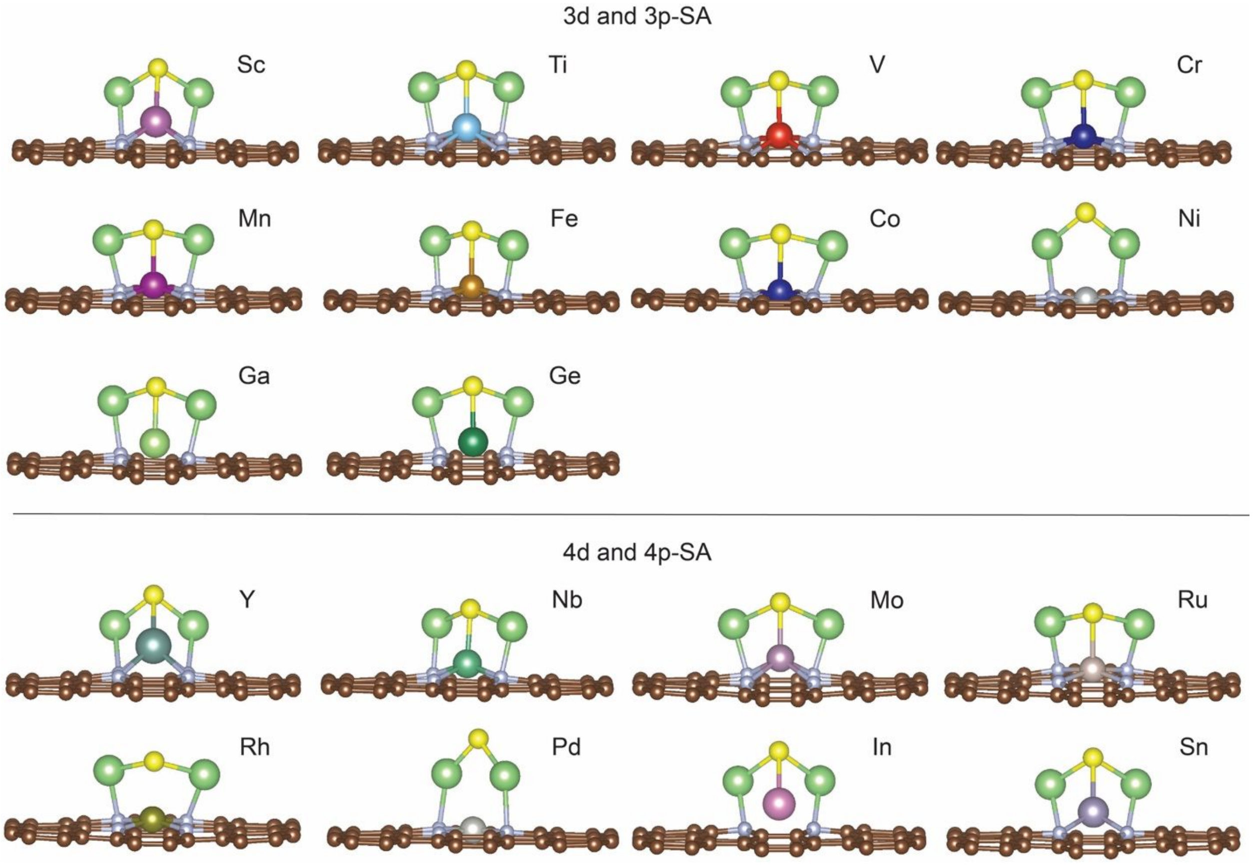

Figure S1. Calculated adsorption structures of Li_2S on metal-SA/NG. The Li, S, C, O, and H atoms are colored green, yellow, brown, red, and white, respectively.

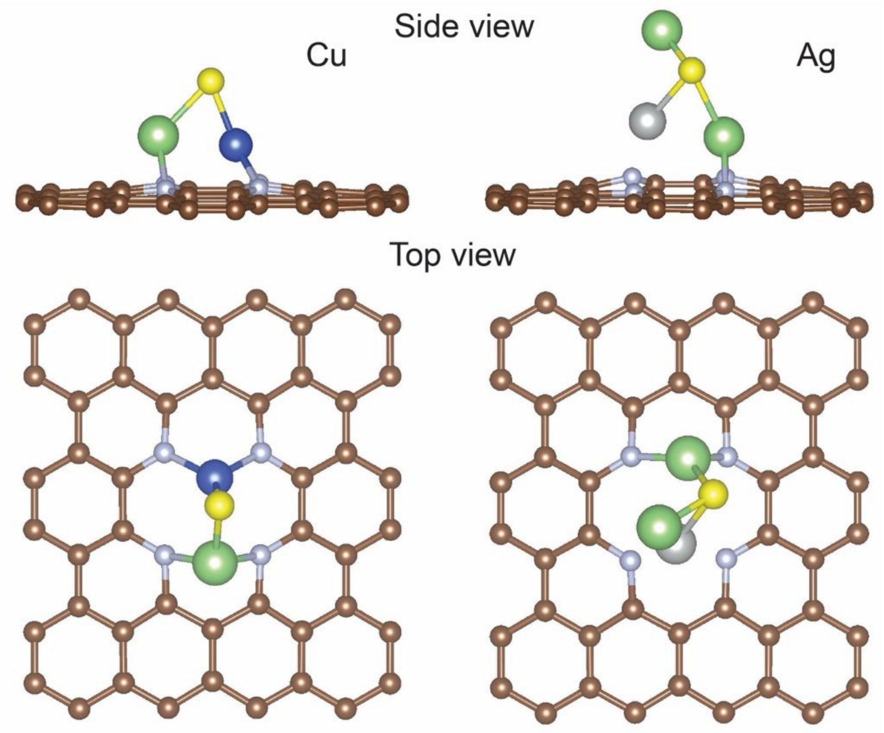

Figure S2. The deformed structures after adsorption of LiS on Cu-SA/NG and Li_2S on Ag-SA/NG. Due to this large deformation, both metal-SAC are not included in the discussion of thermodynamics and kinetics of the charging/discharging of LiS.

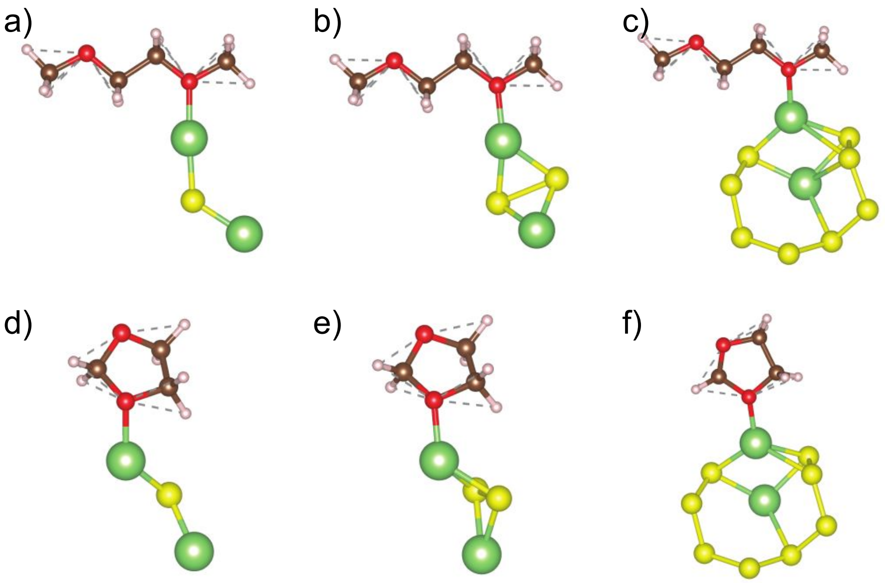

Figure S3. Optimized adsorption structures of Li_2S, Li_2S_2 and Li_2S_8 with electrolyte DME and DOL molecules. The Li, S, C, O, and H atoms are colored green, yellow, brown, red, and white, respectively.

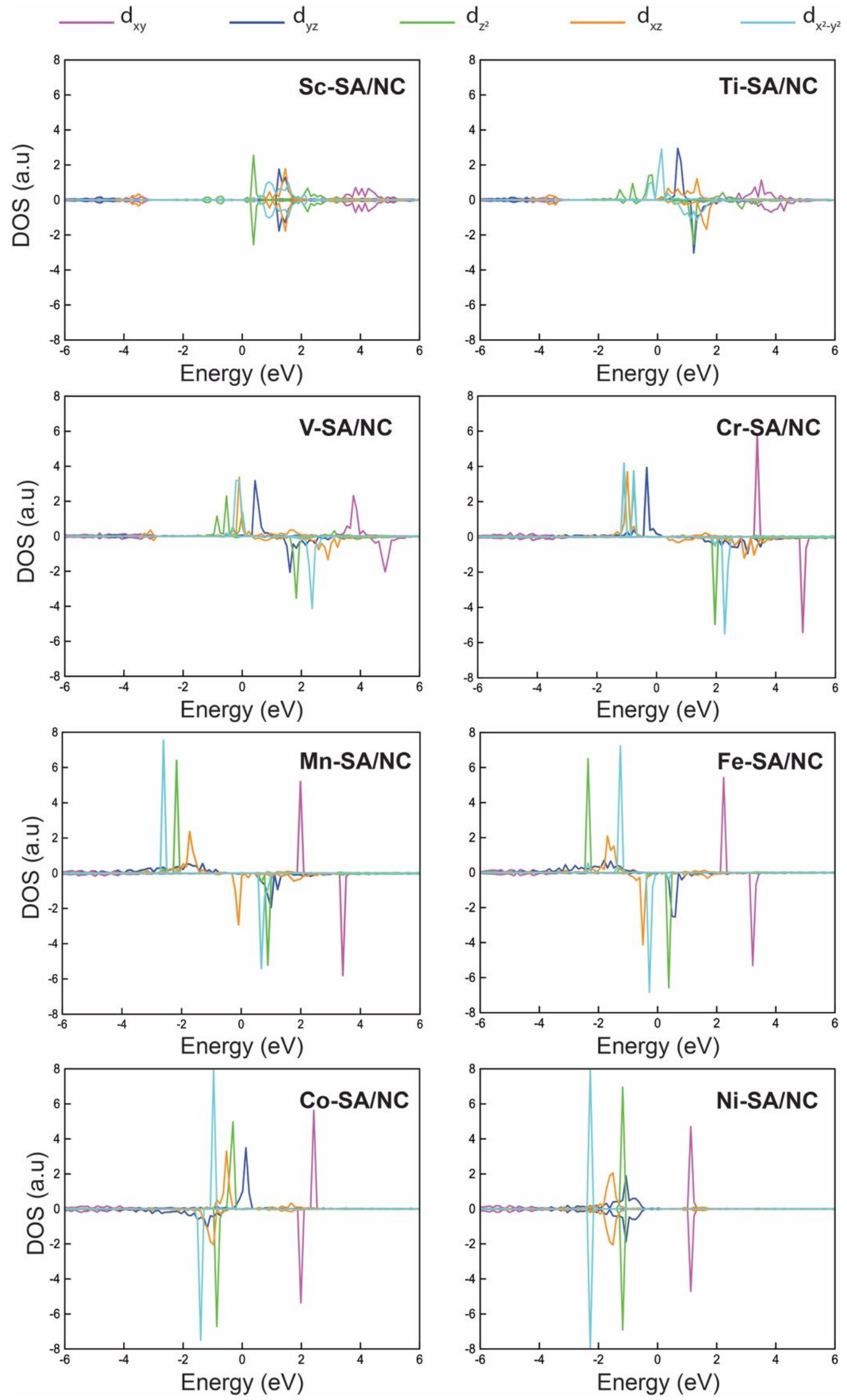

Figure S4. Projected DOS onto d orbitals of M-SA/NG (M is 3d transition metal).

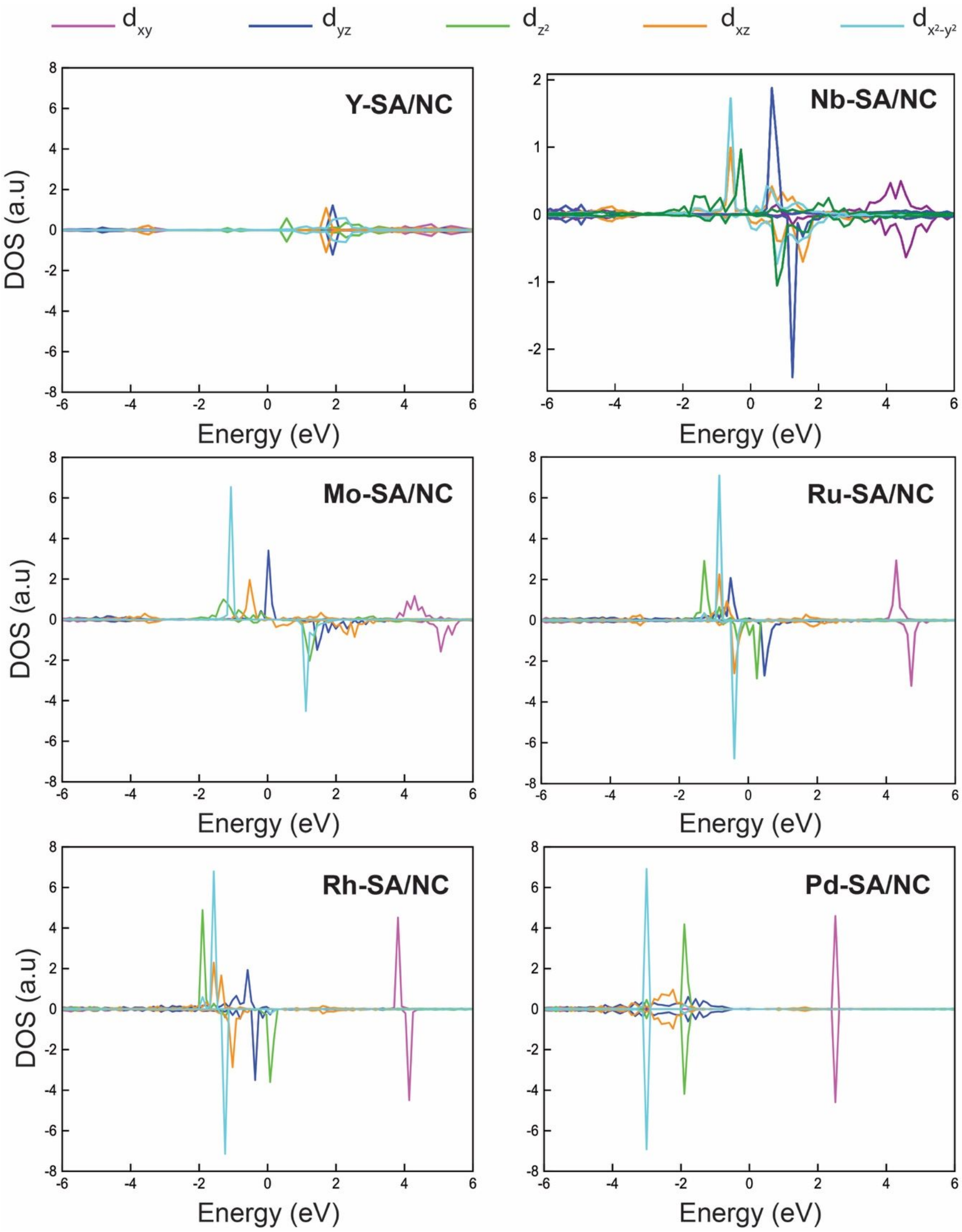

.

Figure S5. Projected DOS onto d orbitals of M-SA/NG (M is 4d transition metal).

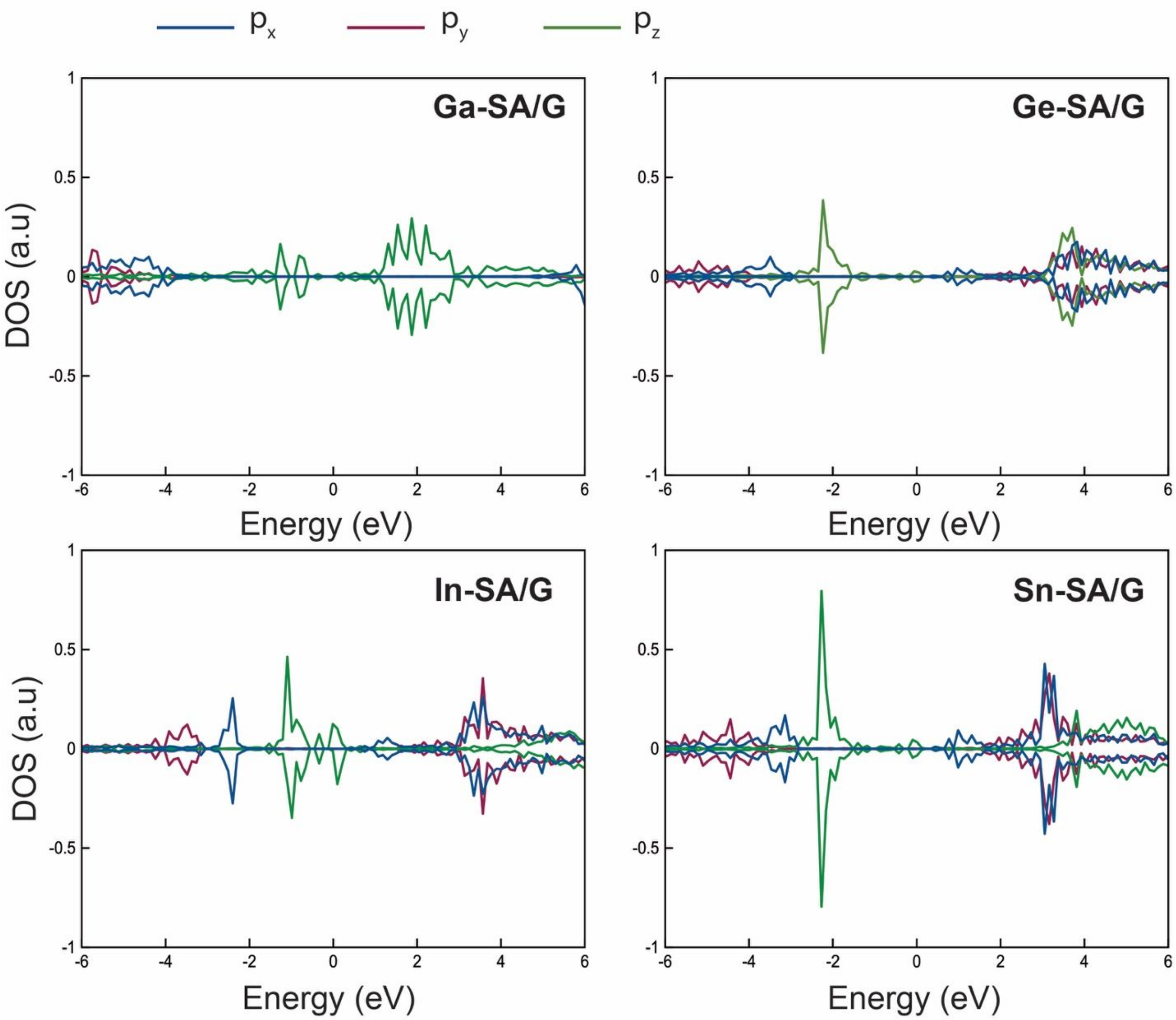

Figure S6. Projected DOS onto p orbitals of M-SA/NG in group 13 (Ga, In) and group 14 (Ge, Sn)

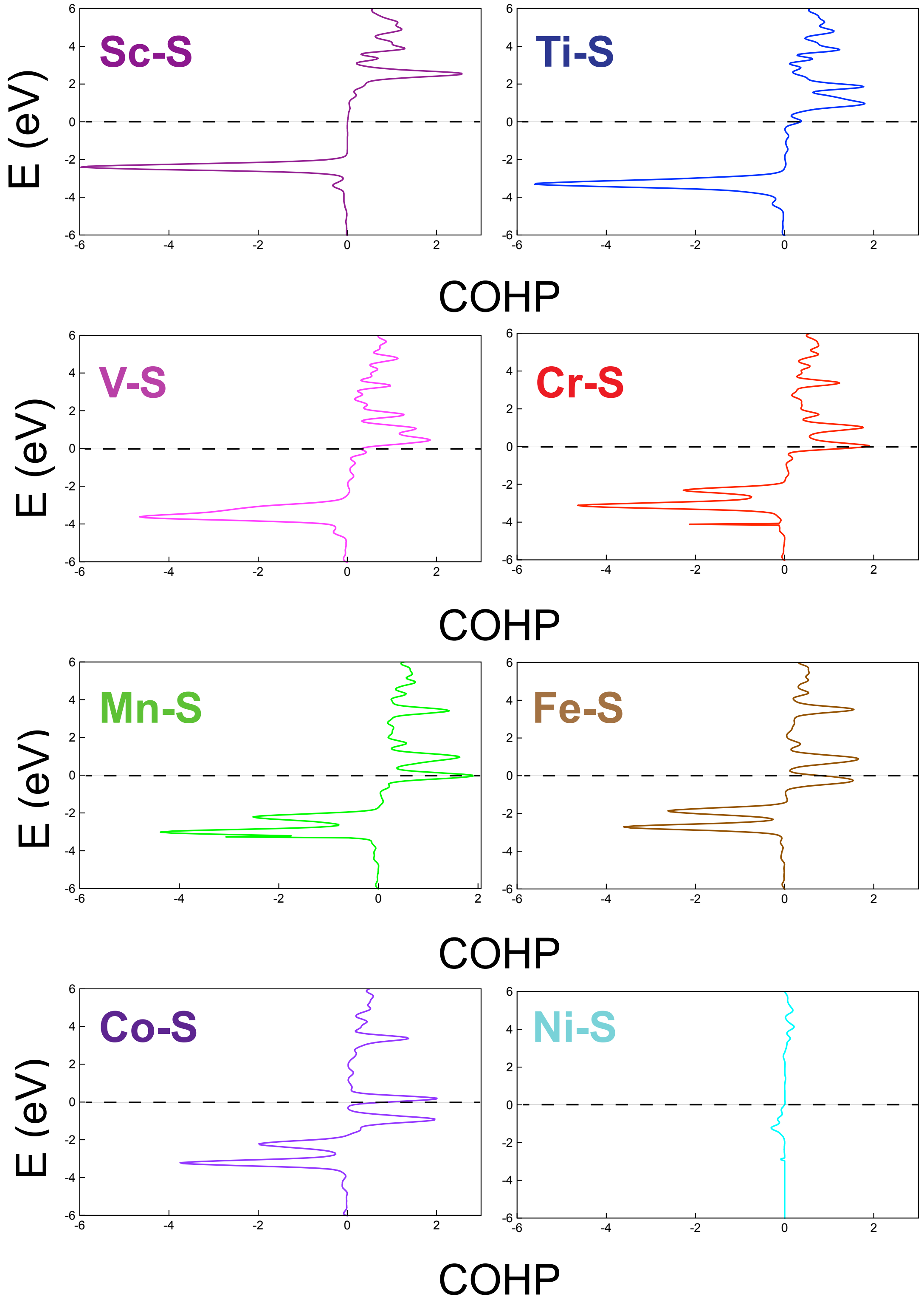

Figure S7. COHP of M-SA/NG (M is 3d transition metal).

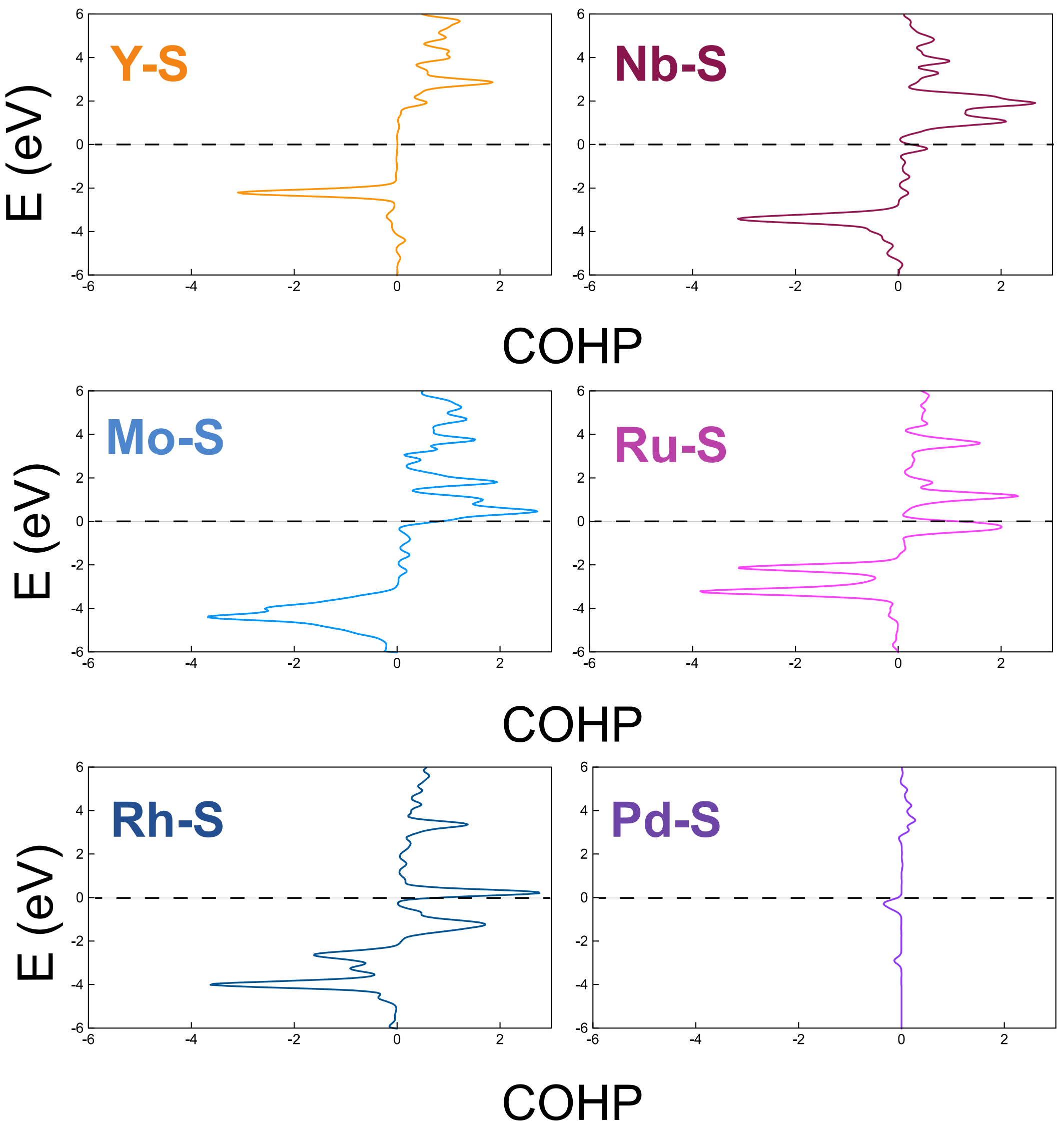

Figure S8. COHP of M-SA/NG (M is 4d transition metal).

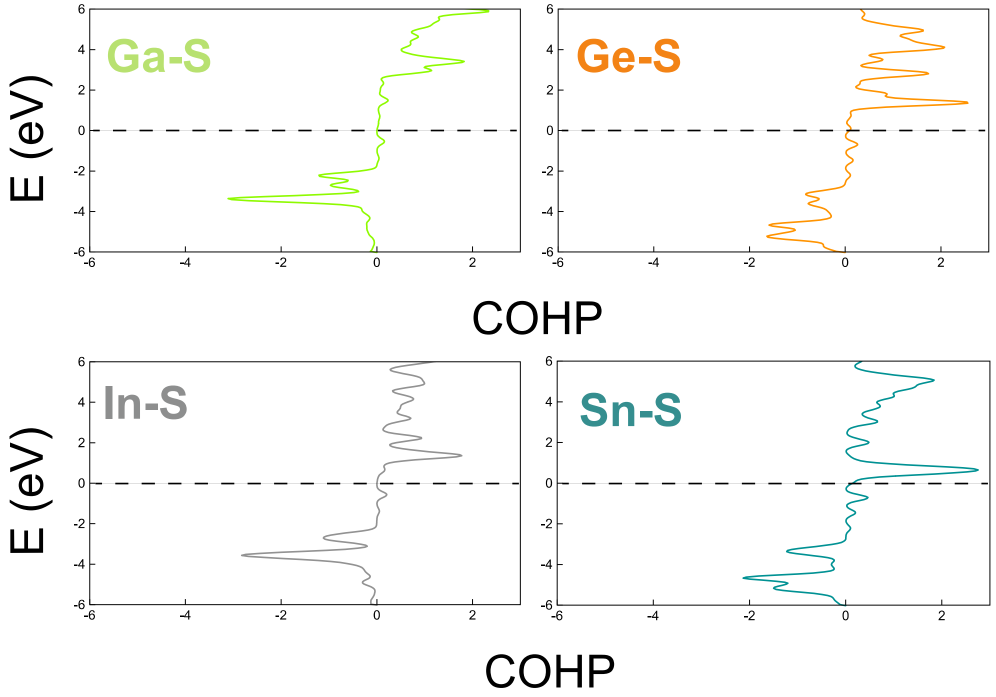

Figure S9. COHP of M-SA/NG of metal in group 13 (Ga, In) and group 14 (Ge, Sn)

their conversion reactions.

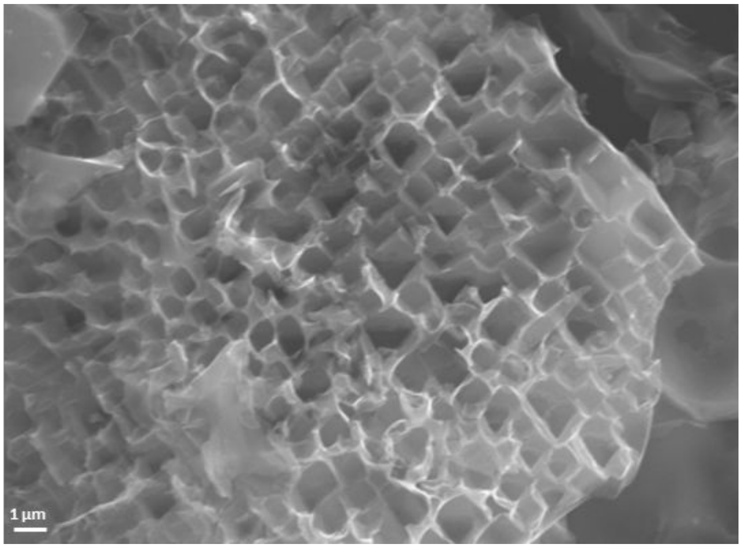

Figure S10. SE SEM image of NC.

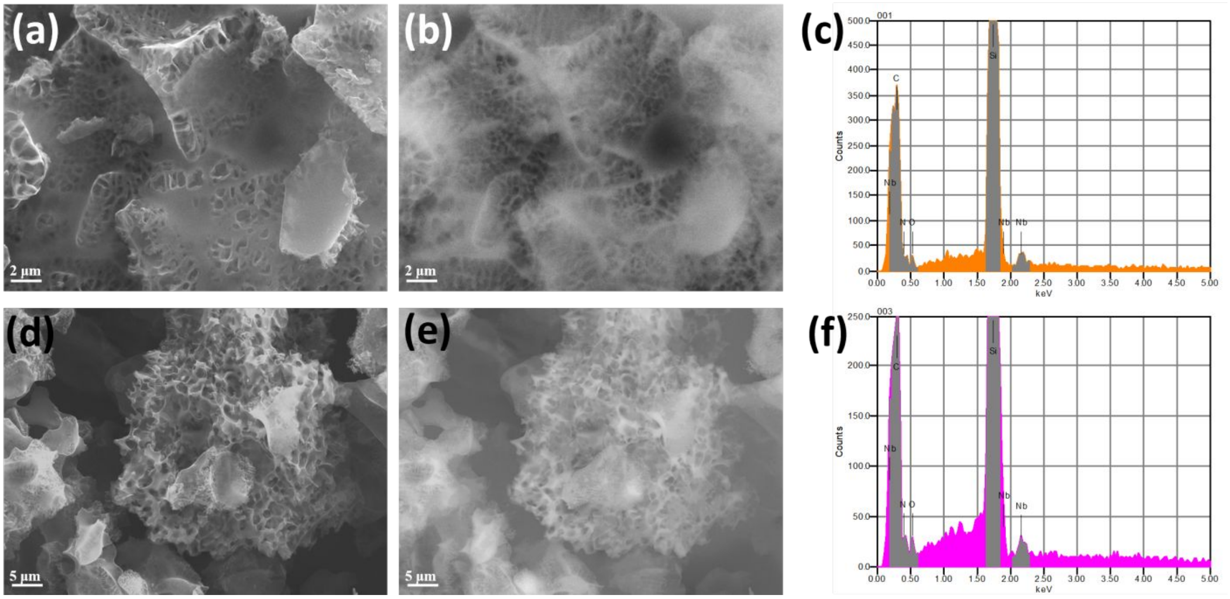

Figure S11. (a), (d) SE and (b), (e) BSE SEM images of Nb-SA/NC and the (c), (f) corresponding EDS spectra for the regions depicted in the images.

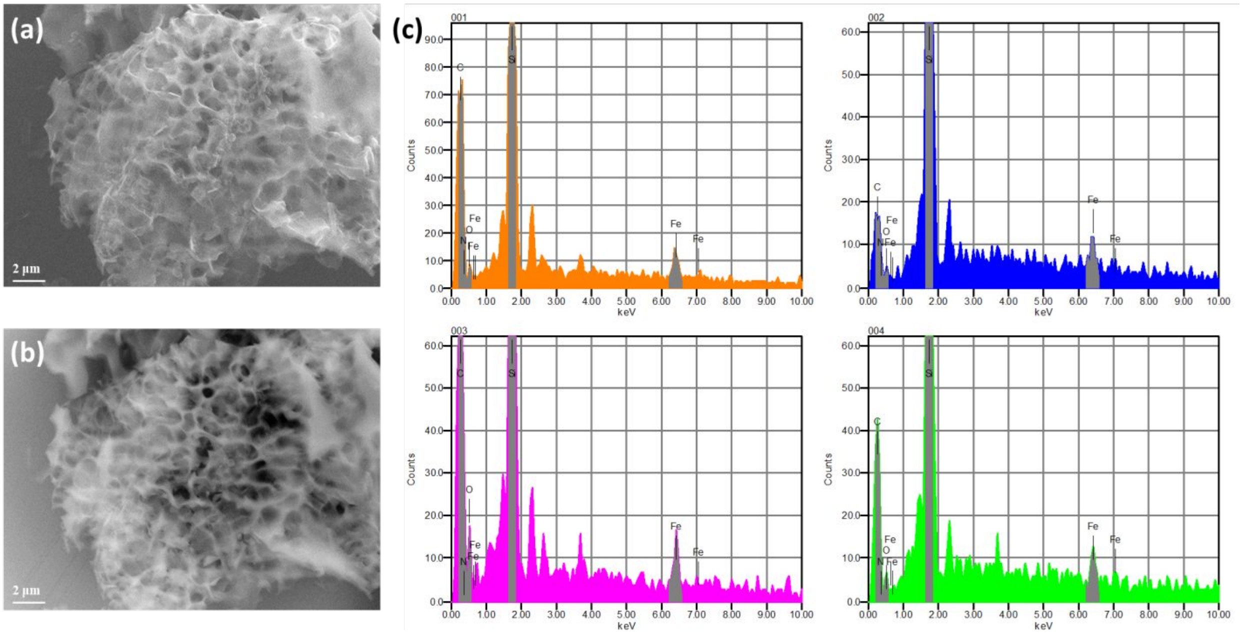

Figure S12. (a) SE and (b) BSE SEM images of Fe-SA/NC and the (c) corresponding EDS spectra for the regions depicted in the images.

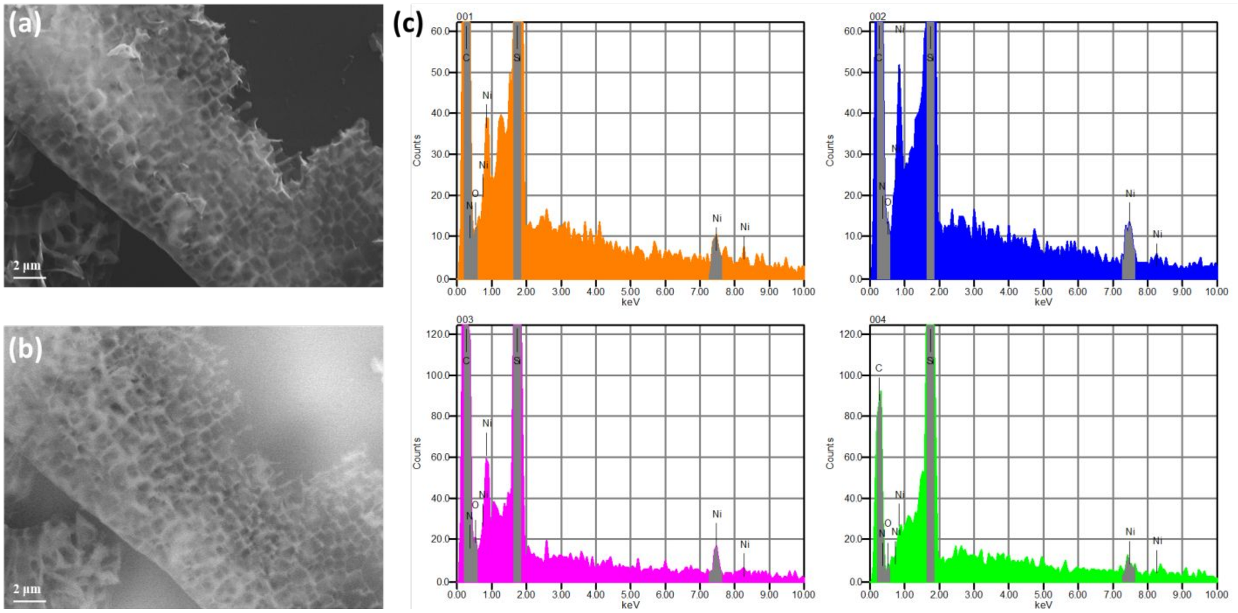

Figure S13. (a) SE and (b) BSE SEM images of Ni-SA/NC and the (c) corresponding EDS spectra for the regions depicted in the images.

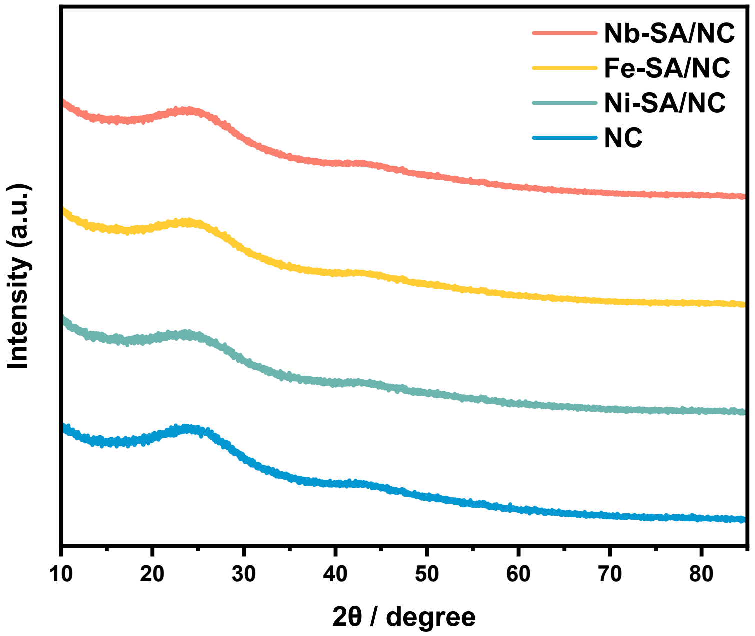

Figure S14. XRD pattern of Nb-SA/NC, Fe-SA/NC, Ni-SA/NC and NC.

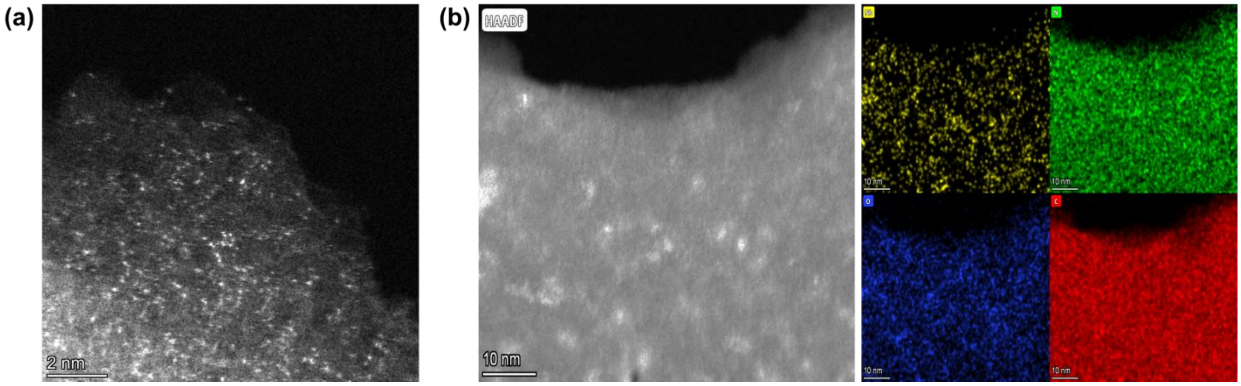

Figure S15. (a) Atomic-resolution aberration-corrected HAADF–STEM image of Nb-SA/NC. (b) HAADF–STEM image of Nb-SA/NC with its corresponding elemental maps showing the distribution of Nb (yellow), N (green), O (blue), and C (red).

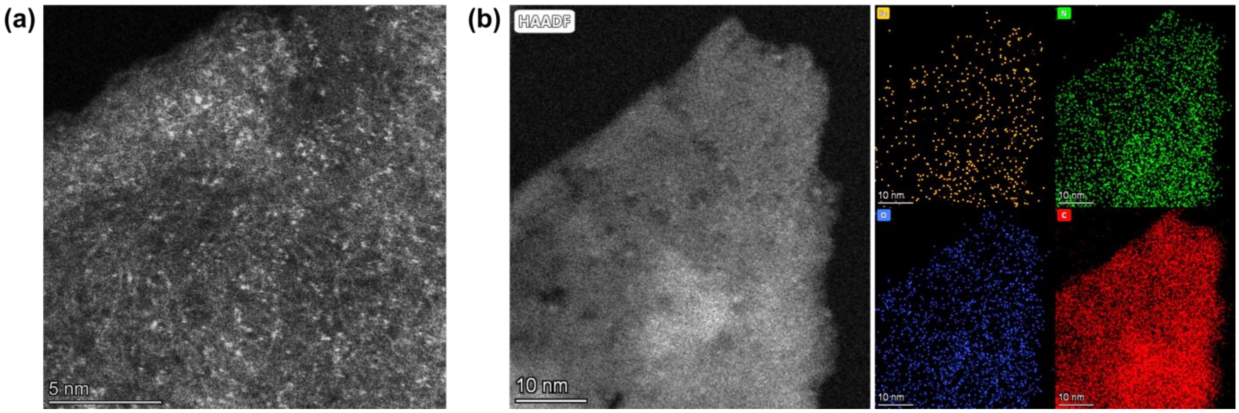

Figure S16. (a) Atomic-resolution aberration-corrected HAADF–STEM image of Fe-SA/NC. (b) HAADF–STEM image of Fe-SA/NC with its corresponding elemental maps showing the distribution of Fe (yellow), N (green), O (blue), and C (red).

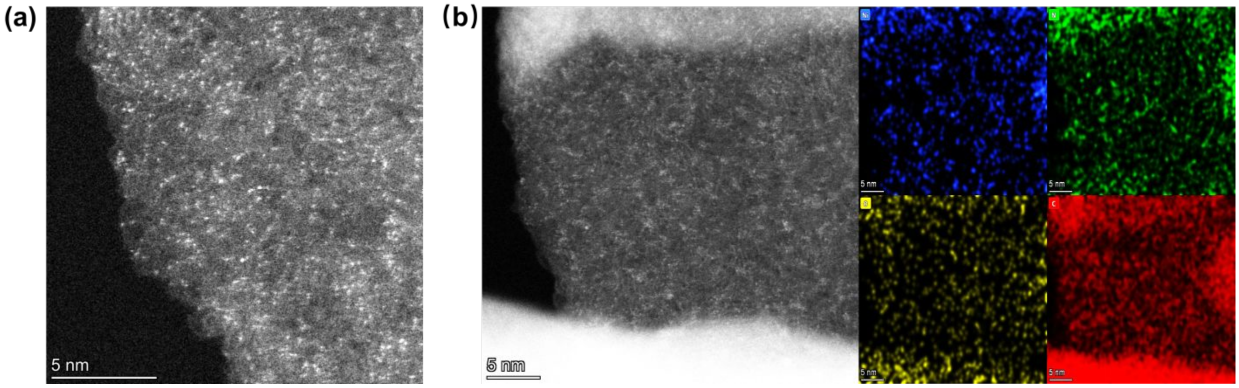

Figure S17. (a) Atomic-resolution aberration-corrected HAADF–STEM image of Ni-SA/NC. (b) HAADF–STEM image of Ni-SA/NC with its corresponding elemental maps showing the distribution of Ni (blue), N (green), O (yellow), and C (red).

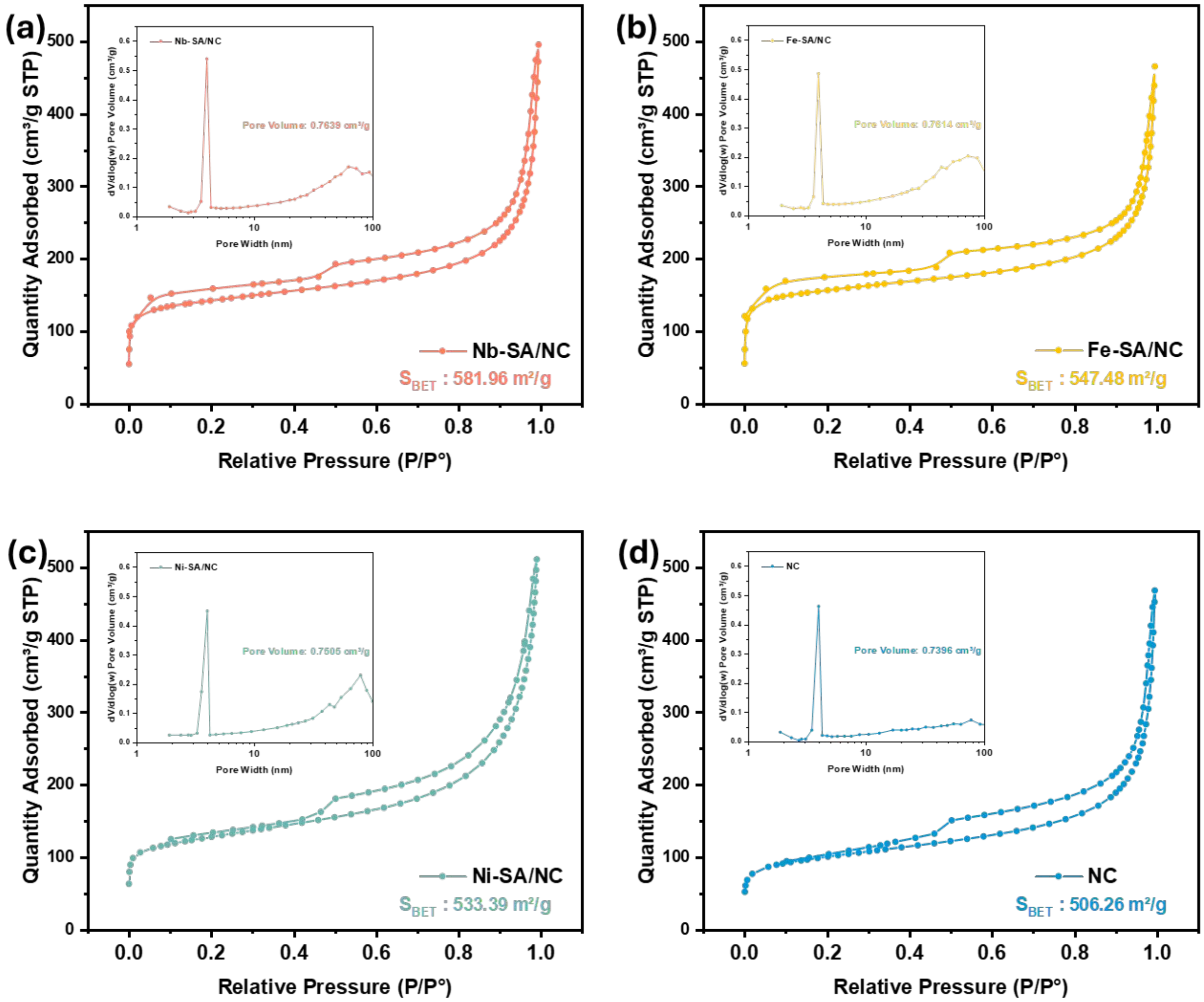

Figure S18. The N_2 adsorption/desorption isotherms and pore size distribution (inset) of (a) Nb-SA/NC, (b) Fe-SA/NC, (c) Ni-SA/NC and (d) NC samples.

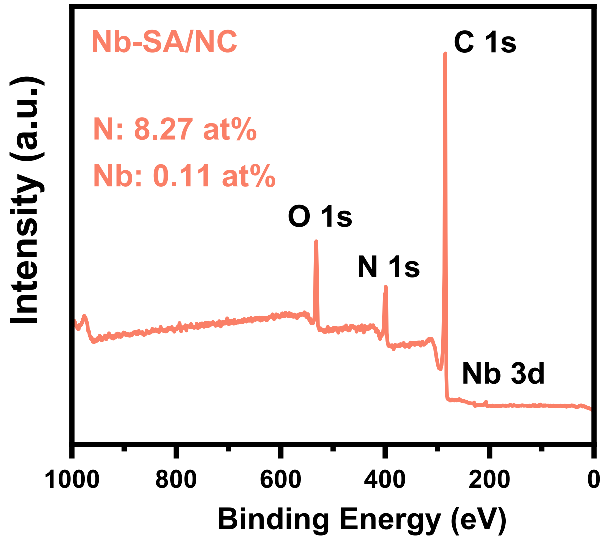

Figure S19. XPS wide survey of Nb-SA/NC.

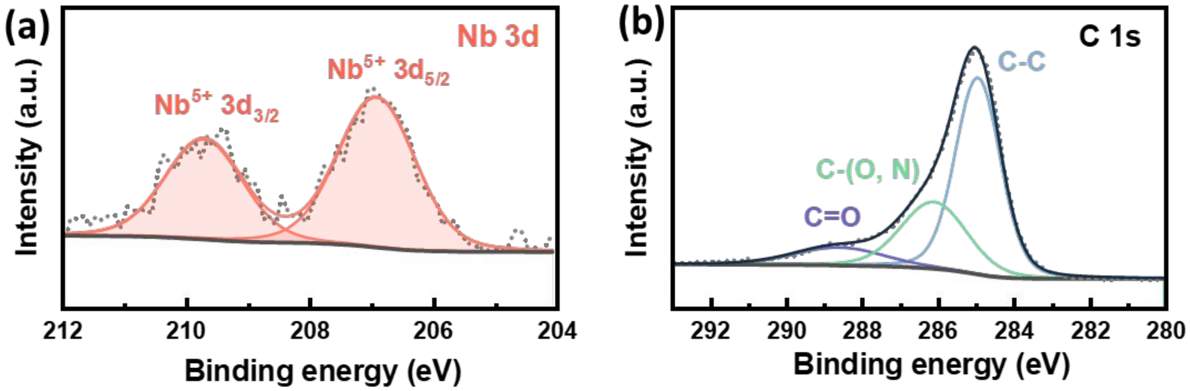

Figure S20. (a) Nb 3d, and (b) C 1s XPS spectra of Nb-SA/NC.

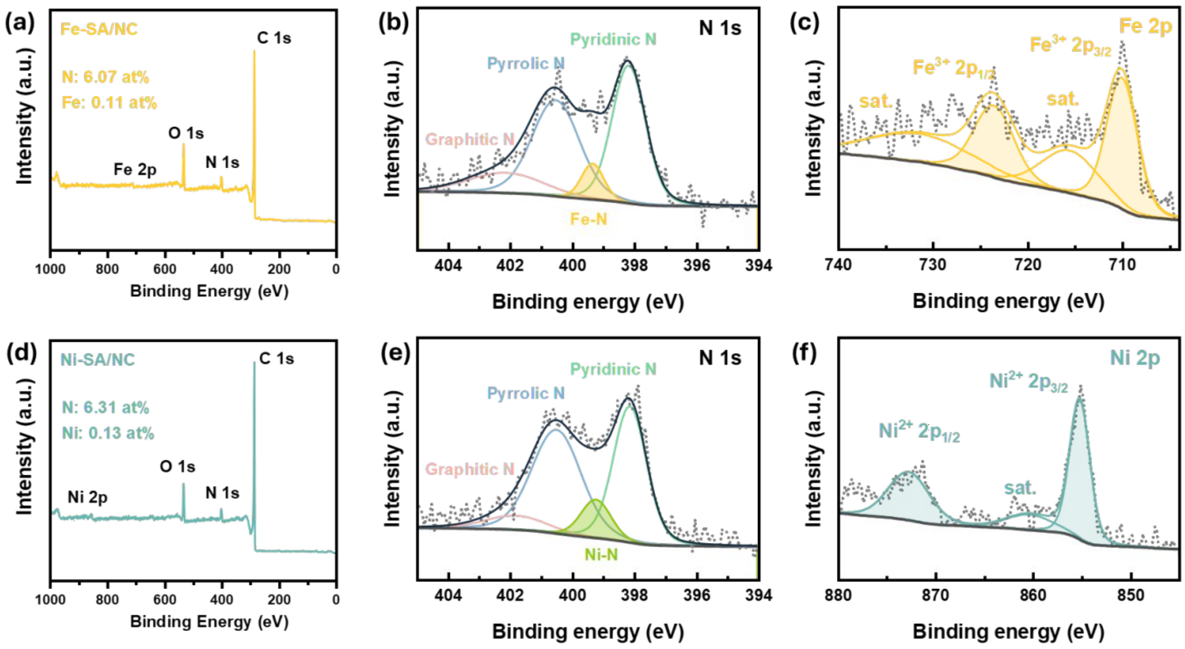

Figure S21. (a) XPS wide survey, (b) N 1s, and (c) Fe 2p XPS spectra of Fe-SA/NC. (d) XPS wide survey, (e) N 1s, and (f) Ni 2p XPS spectra of Ni-SA/NC.

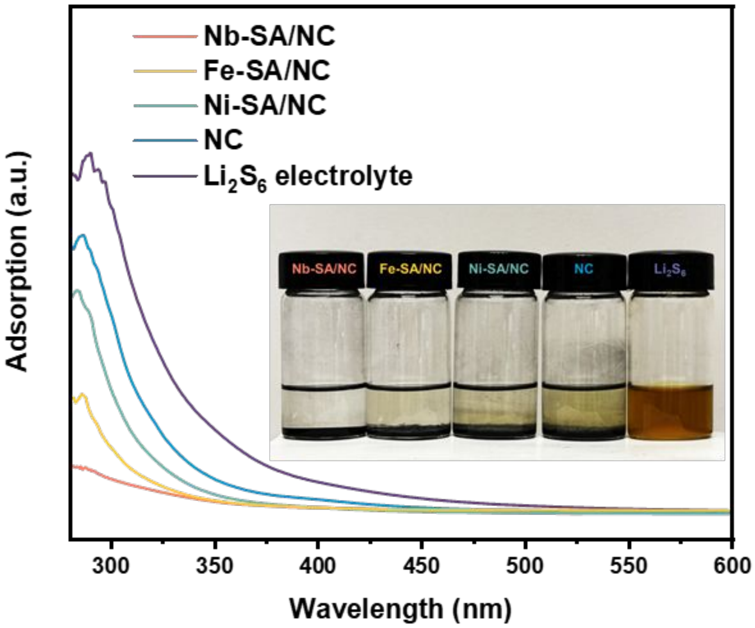

Figure S22. UV–Vis absorbance spectra and digital photos (inset) of 0.005 M Li_2S_6 solution and Li_2S_6 solutions after adsorption tests with different catalyst samples.

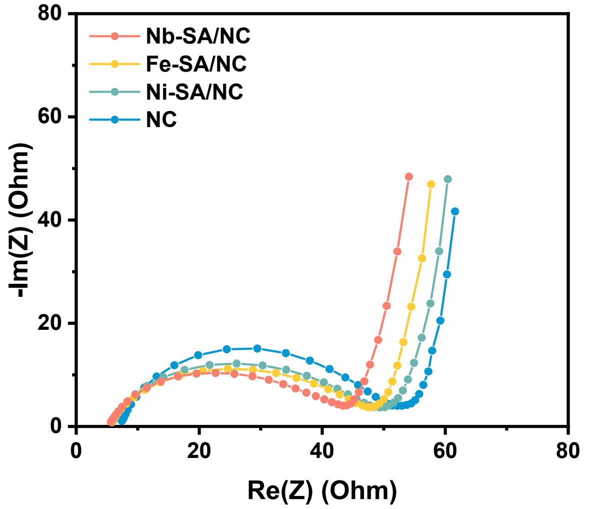

Figure S23. EIS Nyquist plots of Li–S full cells with Nb-SA/NC, Fe-SA/NC, Ni-SA/NC and NC-based sulfur cathodes.

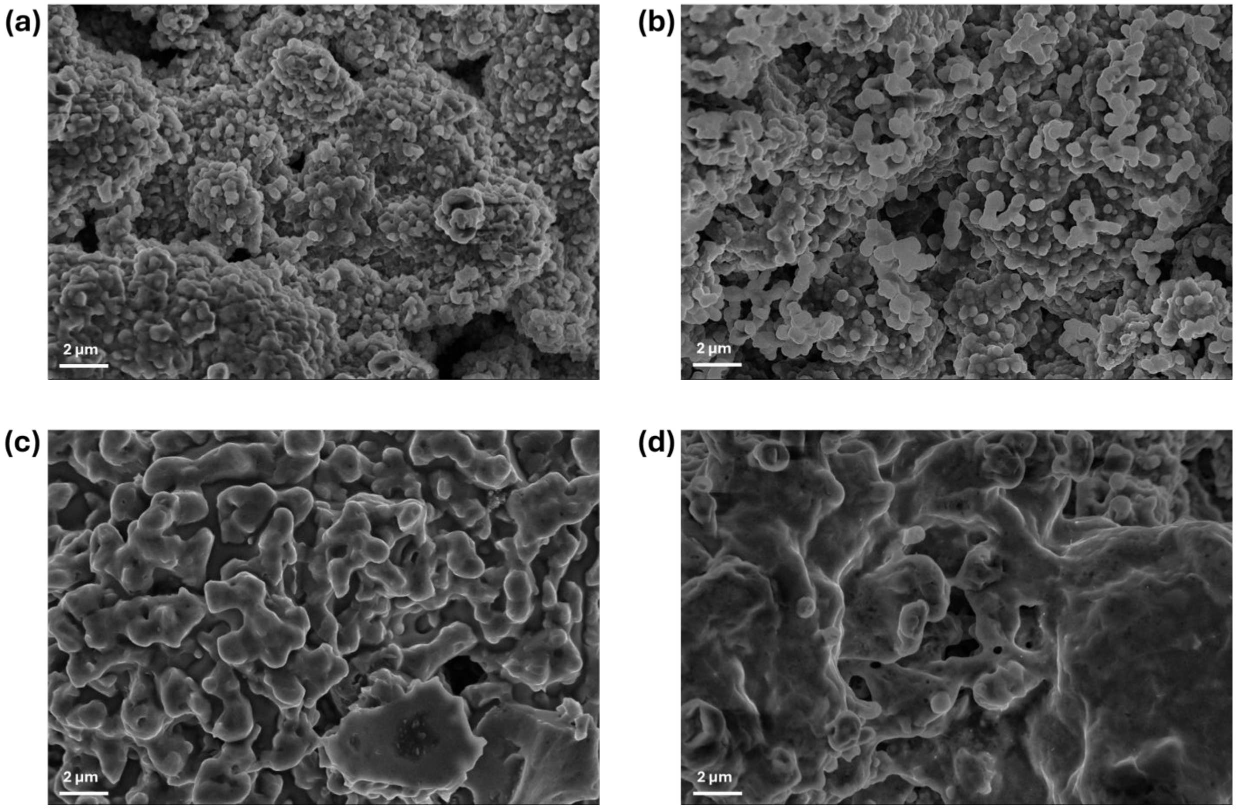

Figure S24. SEM images showing the Li_2S precipitation morphology on (a) Nb–SA/NC, (b) Fe–SA/NC, (c) Ni–SA/NC, and (d) NC.

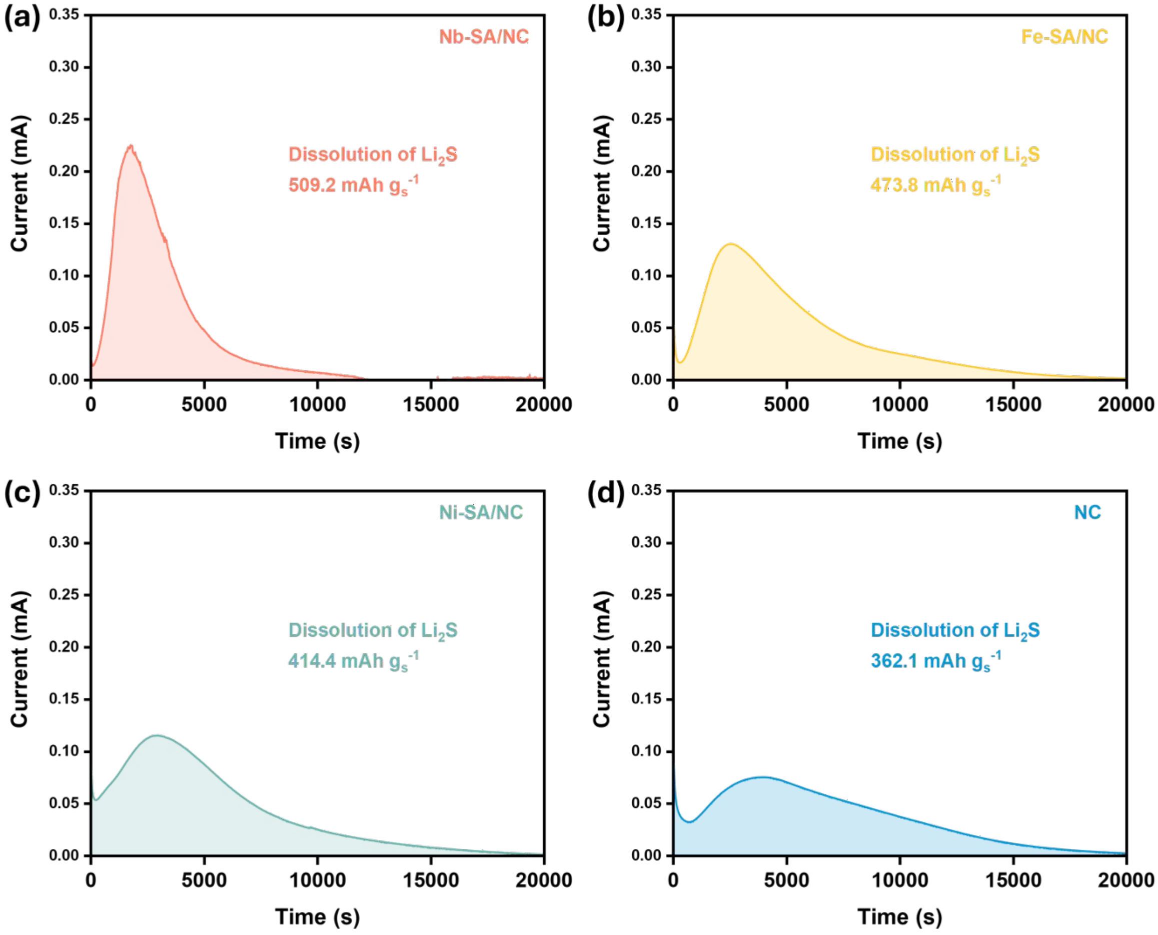

Figure S25. Potentiostatic dissolution profiles of Li_2S on (a) Nb-SA/NC, (b) Fe-SA/NC, (c) Ni-SA/NC, and (d) NC electrodes.

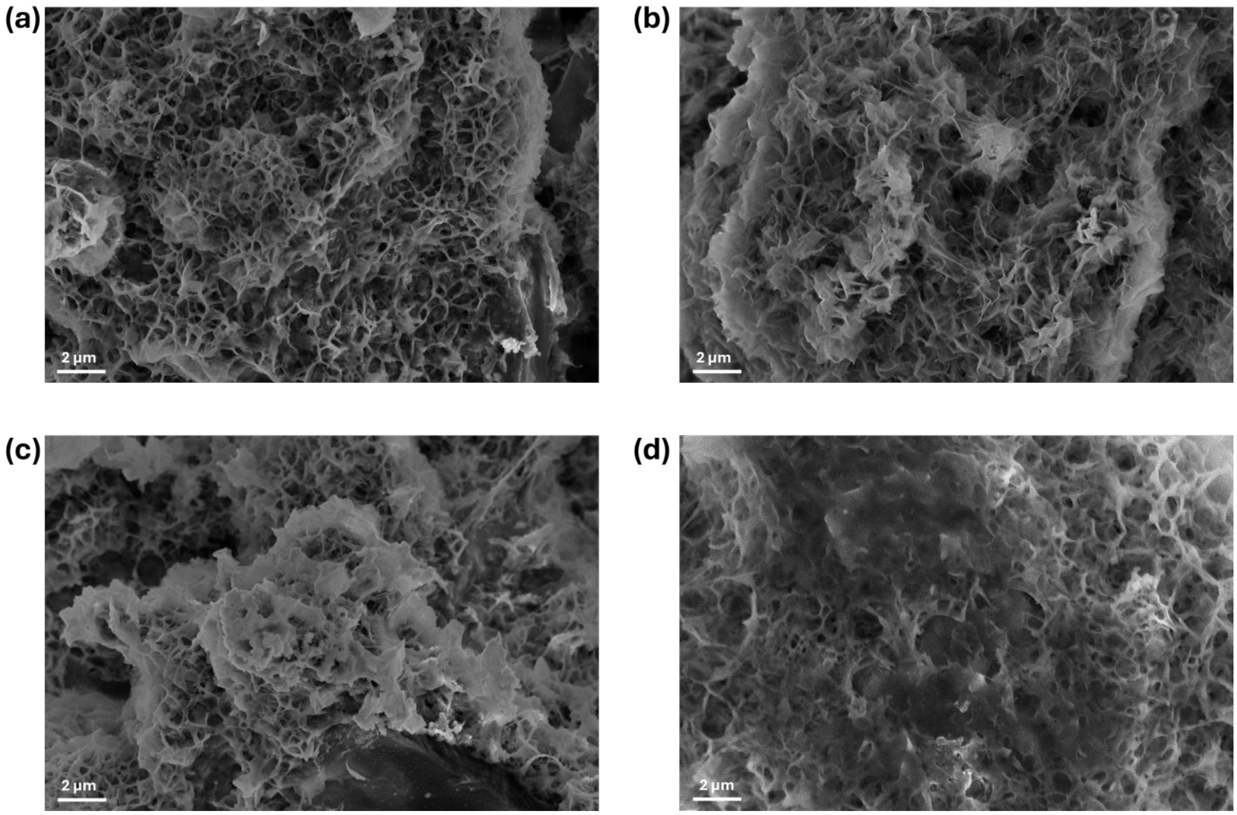

Figure S26. SEM images showing the Li_2S dissociation morphology on (a) Nb-SA/NC, (b) Fe-SA/NC, (c) Ni-SA/NC, and (d) NC.

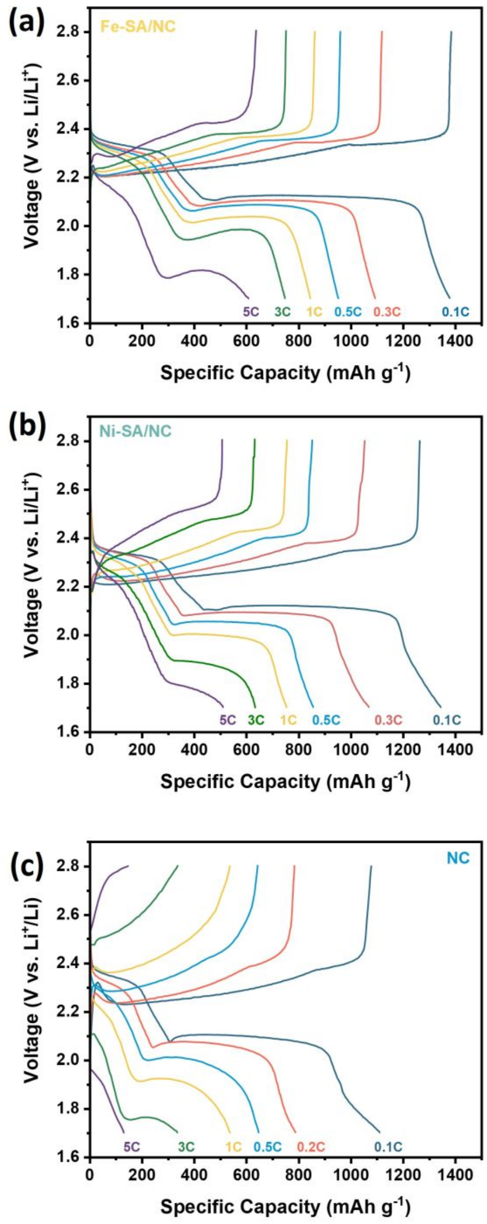

Figure S27. Galvanostatic charge and discharge curves of Li-S cells assembled with Fe-SA/NC, Ni-SA/NC and NC-based cathode.

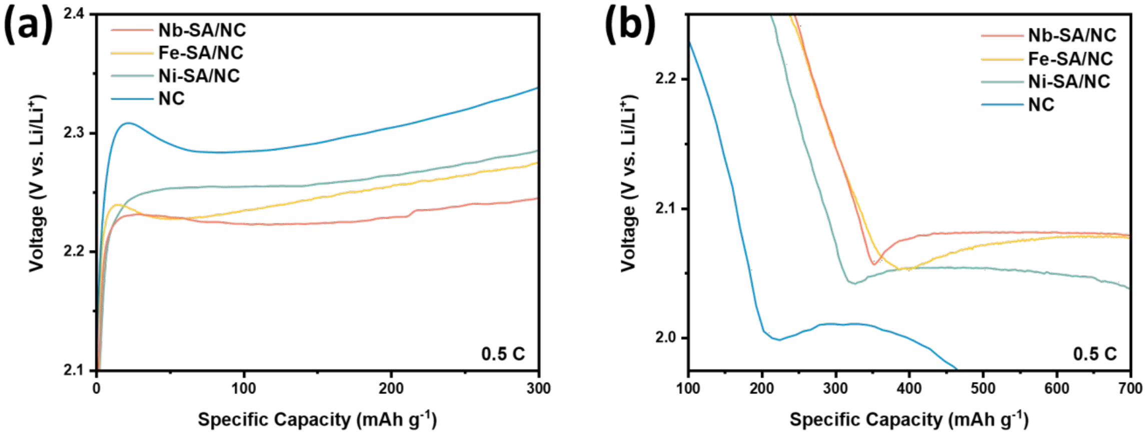

Figure S28. First (a) charge and (b) discharge voltage profile comparison between Nb-SA/NC, Fe-SA/NC, Ni-SA/NC and NC at 0.5C.

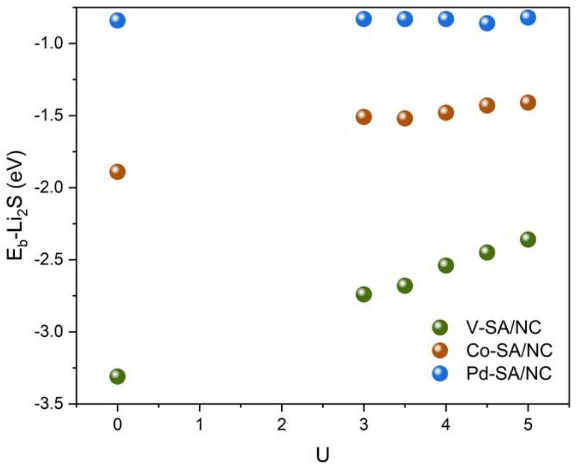

Figure S29. Li_2S adsorption for testing on V-SA/NC, Co-SA/NC and P-SA/NC, calculated at the GGA+U Level.

Table S1. The integral of the negative COHP up to the Fermi level (ICOHP)

	**Metal-S**	**Li1-N1**	**Li2-N2**	**sum**
**Sc**	4.93	1.02	1.03	6.98
**Y**	4.03	1.15	1.10	6.28
**Ti**	5.59	1.21	1.21	8.01
**V**	5.89	1.3	1.3	8.17
**Nb**	4.95	2.17	2.16	9.28
**Cr**	5.00	1.07	1.07	7.13
**Mo**	7.16	1.14	1.13	9.43
**Mn**	4.76	1.12	1.11	6.99
**Fe**	3.88	1.15	1.15	5.72
**Ru**	4.47	1.59	1.58	7.64
**Co**	3.41	0.80	1.04	5.25
**Rh**	3.79	0.89	1.13	5.81
**Ni**	0.3	1.02	1.05	2.37
**Pd**	0.3	0.98	1.05	2.33

Table S2. The reaction energy of the two elementary steps studied in the main text, with and without including the solvent molecules.

	Vacuum		Solvation	
	**ΔG_1 (eV)**	**ΔG_2 (eV)**	**$\Delta G'_1$ (eV)**	**$\Delta G'_2$ (eV)**
**Sc**	2.76	3.76	3.55	4.86
**Y**	2.81	3.70	3.68	4.85
**Ti**	2.3	2.58	3.21	3.68
**V**	2.39	2.42	3.42	3.55
**Nb**	2.04	2.51	2.94	3.65
**Cr**	2.84	2.69	3.81	3.75
**Mo**	2.19	2.36	2.81	3.49
**Mn**	2.77	2.78	3.64	3.84
**Fe**	2.91	2.83	3.81	3.85
**Ru**	2.95	2.91	3.80	3.95
**Co**	3.09	3.23	4.57	3.61
**Rh**	2.99	3.27	3.75	4.28
**Ni**	3.37	3.35	4.31	4.19
**Pd**	3.43	3.42	4.30	4.41
**Ga**	2.66	3.96	3.71	4.77
**In**	2.50	3.61	3.36	4.96
**Ge**	2.15	2.73	3.24	3.85
**Sn**	2.05	2.63	2.88	3.74

Table S3. The Bader Charges Carried by single atom on SA/NC surface, calculated at the GGA+U Level.

	Charge |e|		
**U**	**V**	**Co**	**Pd**
0.0	1.32	0.87	0.73
3.0	1.81	1	0.91
3.5	1.81	1.15	0.9
4.0	1.83	1.1	0.88
4.5	1.81	1.12	0.86
5.0	1.72	1.28	0.84

Table S4. Quantification results of XPS analysis for Nb-SA/NC, Fe-SA/NC and Ni-SA/NC.

Sample	Metal at%	N at%	Pyridinic N in total N (%)	Pyrrolic N in total N (%)	Graphitic N in total N (%)	M-N bond in total N (%)	Ratio of M-N to M
Nb-SA/NC	0.11	8.27	45.17	36.31	13.4	5.12	3.85
Fe-SA/NC	0.11	6.07	40.35	39.90	12.93	6.82	3.75
Ni-SA/NC	0.13	6.31	43.79	38.90	9.47	7.84	3.81
